# The Role of Idiothetic Signals, Landmarks, and Conjunctive Representations in the Development of Place and Head-Direction Cells: A Self-Organizing Neural Network Model

**DOI:** 10.1093/texcom/tgab052

**Published:** 2021-08-27

**Authors:** Toby St. Clere Smithe, Simon M Stringer

**Affiliations:** Department of Experimental Psychology, Centre for Theoretical Neuroscience and Artificial Intelligence, University of Oxford, Oxford OX2 6NW, UK; Department of Experimental Psychology, Centre for Theoretical Neuroscience and Artificial Intelligence, University of Oxford, Oxford OX2 6NW, UK

**Keywords:** attractor networks, developmental neurobiology, head-direction cell, path integration, place cell

## Abstract

Place and head-direction (HD) cells are fundamental to maintaining accurate representations of location and heading in the mammalian brain across sensory conditions, and are thought to underlie path integration—the ability to maintain an accurate representation of location and heading during motion in the dark. Substantial evidence suggests that both populations of spatial cells function as attractor networks, but their developmental mechanisms are poorly understood. We present simulations of a fully self-organizing attractor network model of this process using well-established neural mechanisms. We show that the differential development of the two cell types can be explained by their different idiothetic inputs, even given identical visual signals: HD cells develop when the population receives angular head velocity input, whereas place cells develop when the idiothetic input encodes planar velocity. Our model explains the functional importance of conjunctive “state-action” cells, implying that signal propagation delays and a competitive learning mechanism are crucial for successful development. Consequently, we explain how insufficiently rich environments result in pathology: place cell development requires proximal landmarks; conversely, HD cells require distal landmarks. Finally, our results suggest that both networks are instantiations of general mechanisms, and we describe their implications for the neurobiology of spatial processing.

## Introduction

### Spatial Representations in the Rat Brain

The rat brain contains a variety of different types of cells that represent the position and orientation of the animal within its environment.

Head-direction (HD) cells, originally found in rats, fire maximally when the animal’s head is facing in a particular preferred direction (Ranck 1984; Taube et al. 1990; Muller et al. 1996; Taube et al. 1996). HD cell activity constitutes an animal’s internal representation of its planar heading, forming a one-dimensional population code that is commonly modeled as a continuous attractor neural network (CANN) (Skaggs et al. 1995; Zhang 1996; Stringer, Trappenberg, et al. 2002b; Wiener and Taube 2005; Stringer and Rolls 2006; Walters and Stringer 2010; Clark and Taube 2012). The HD signal was initially observed in the postsubiculum (Ranck 1984), and subsequent experimental work traced its origins through a network of limbic structures to the lateral mammillary nucleus (LMN) (Sharp, Blair, et al. 2001a). An important property of HD cells is that they are able to update their firing using idiothetic (self-motion) signals as the animal rotates in the absence of external sensory input—a property known as “path integration” (Taube et al. 1996). The rat HD system is known to receive idiothetic inputs from angular head velocity (AHV) cells (Bassett and Taube 2001; Bassett et al. 2007). These idiothetic signals are thought to provide the self-motion signals needed for path integration of HD in the dark. Other cells found in the rat HD system represent a conjunction of HD and AHV (Chen et al. 1994; Sharp 1996; Stackman and Taube 1998; Sharp, Blair, et al. 2001a; Bassett and Taube 2005; Jercog et al. 2019). It has been posited that such conjunction cells may play an important intermediary role in path integration (Stringer and Rolls 2006; Walters and Stringer 2010; Walters et al. 2013; Page et al. 2018).

Place cells are also observed in the limbic system, being typically reported when principal cells of hippocampal CA3 or CA1 fields are found to have place-specific activity (O'Keefe and Dostrovsky 1971; O'Keefe 1976). Like the HD representation, the place representation forms a population code, commonly modeled using CANNs (Battaglia and Treves 1998; Stringer, Rolls, et al. 2002a; Stringer, Trappenberg, et al. 2002b; Corneil and Gerstner 2015; Steemers et al. 2016), though in this case distributed over a two-dimensional manifold. This representation persists even in the absence of external sensory cues, such as in the dark, tracking the animal’s location in its internal map (Muller and Kubie 1987; O'Keefe and Speakman 1987; Quirk et al. 1990; Markus et al. 1994)—a form of path integration. As with the HD system, the hippocampus is known to receive the relevant idiothetic signals to perform this task—forward motion and HD (Leutgeb et al. 2000; Stackman et al. 2002)—and indeed there is evidence for conjunction neurons encoding combinations of forward motion, HD and place (Leutgeb et al. 2000; Sargolini 2006; Lu and Bilkey 2009; Chen et al. 2012).

The responses of both HD cells and place cells are largely anchored to visual and other external sensory cues (Diba and Buzsaki 2008), but can be sustained and updated in the absence of external sensory input using only idiothetic (self-motion) signals available in the brain (McNaughton et al. 2006).

The HD cell and place cell systems appear to operate synergistically in the brain to support navigation. For example, disrupting the HD system leads to perturbed place representations (Stackman et al. 2002), as well as path-integration failure (Golob and Taube 1999). Similarly, disruptions to the hypothesized place cell networks lead to navigational difficulties (Morris et al. 1982).

Owing to their anatomical and functional proximity, as well as their similar modeling history, it is natural to hypothesize that the two networks are instances of a general principle underlying spatial learning and memory in the brain. Computationally, each system has the form of a state-space model (Chen and Brown 2013), in which the changing value of a state, such as heading or location, is inferred from the current state and observed signals such as visual, tactile, olfactory, and idiothetic inputs; for simplicity, we consider in this work only one type of external sensory input, that we call “visual,” but it is idealized and could be replaced with another input type without affecting the results obtained.

The key questions addressed in this paper are therefore as follows. How might a common form of neural network architecture give rise to either HD cells or place cells, especially given their shared input from the sensory environment? Exactly what determines whether the network develops HD cells or place cells? And how could these computational mechanisms be implemented in a biologically plausible manner, using a plausible network architecture and synaptic learning rules?

### The Development of HD Cells and Place Cells—A Hypothesis

We propose that the different firing properties of HD cells and place cells in the rat brain arise because the different brain areas containing these two classes of cells receive distinct kinds of idiothetic (self-motion) signal. Specifically, the rat HD system receives inputs from idiothetic cells representing AHV, while place cells in the rat hippocampus receive idiothetic signals from cells representing a combination of forward motion andHD.

Let us consider what may happen in the rat HD system during visually guided learning as the animal moves about within its environment in the light. Assume that the environment contains a mixture of distal (distant) and proximal (nearby) visual cues. The rat HD system receives idiothetic input signals representing AHV. As the animal moves, the idiothetic AHV signals are always consistent with the egocentric motion of the distal visual cues within the environment with respect to its eyes. That is, when the animal is not rotating, there is no AHV signal and the distal cues remain static in the egocentric (eye-centered) visual space. Contrariwise, when the animal rotates, the AHV signal is nonzero and the distal cues shift in a consistent manner through the egocentric visual space. For distal visual cues, this consistency between the egocentric visual signals and the AHV signals is robust in that it occurs completely independently of any possible translational motion of the animal through the environment. However, the same is not true for proximal visual cues. For example, if we consider any speed of forward motion through the environment with no head rotation, then the proximal visual cues will shift through the egocentric visual space of the rat at that speed while there is no AHV signal. Hence, it is the motion of distal, but not proximal, visual cues that is consistent with AHV signals as the animal explores its sensory environment. Given this, we hypothesized that in the rat HD system, the visual inputs from the distal cues and AHV self-motion signals would reinforce each other during early visually guided learning in the sensory training environment, and drive the development of synaptic connectivity that ensures the spatial cells are individually tuned to the egocentric locations of particular distal visual cues. Such spatial cells would then exhibit the responses of HD cells.

Let us now consider what may happen in the rat hippocampus during visually guided learning as the animal moves about within its environment in the light. Again assume that the environment contains a mixture of distal and proximal visual cues. The rat hippocampus receives both forward motion signals and HD signals, which are combined multiplicatively by conjunction cells that represent movement in a particular direction. As the animal moves, idiothetic signals that combine forward motion and HD are not consistent with the egocentric (retinotopic) motion of the distal visual cues within the environment. For example, in the case of forward motion at any speed, there is an active idiothetic signal while the distal visual cues do not shift within the egocentric visual space. Contrariwise, during rotation, the distal visual cues shift through the egocentric visual space while there is no idiothetic signal. However, the idiothetic signals that combine forward motion and HD are consistent with the egocentric motion of the proximal visual cues as the animal moves within its environment. For example, if the animal moves forward in a particular direction, there is a nonzero idiothetic signal representing this motion and the proximal visual cues shift through the egocentric visual space correspondingly. Alternatively, if the animal rotates on the spot then there is no change in the place in which the animal is situated and correspondingly no idiothetic signal encoding a conjunction of forward motion and HD. For proximal visual cues, this consistency between the egocentric visual signals and the conjunction of forward motion and HD signals is robust in that it occurs independently of any rotational motion. Hence, it is the motion of proximal, but not distal, visual cues that is consistent with the conjunction of forward motion and HD signals. Given this, we hypothesized that in the rat hippocampus, the visual inputs from the proximal cues and the self-motion signals combining forward motion and HD would reinforce each other during early visually guided learning. This would then drive the development of spatial cells that are tuned to particular places within the environment defined by the egocentric positions of the proximal visual cues (Diba and Buzsaki 2008). Such spatial cells would then exhibit the observed responses of place cells found in the rat hippocampus, and indeed we note the reported existence of such landmark-position-dependent place cells (Renaudineau et al. 2007; Komorowski et al. 2009; Deshmukh and Knierim 2013).

In the simulations presented below, we show that a common neural network model architecture can develop during visually guided training either HD cells if the idiothetic inputs encode AHV, or place cells if the idiothetic inputs encode combinations of forward motion andHD.

### Continuous Attractor Neural Networks

In this paper, we use biologically plausible CANN models to simulate the development of HD cells and place cells. Such architectures have long been established in modeling spatial processing circuits in the brain (Stringer, Rolls, et al. 2002a; Stringer, Trappenberg, et al. 2002b; Wiener and Taube 2005; Stringer and Rolls 2006; Walters and Stringer 2010; Clark and Taube 2012; Corneil and Gerstner 2015; Steemers et al. 2016; Rennó-Costa and Tort 2017). In particular, the present work builds on our earlier two-layer CANN model of HD cells (Page et al. 2018), which learned to perform reasonably accurate path integration. Unlike earlier attractor models, the models presented here are fully self-organizing, using only simple biologically plausible mechanisms.

CANN models explain how a neural population can maintain a short-term memory representation of a continuous-valued state variable, such as an animal’s HD or place, and how such a population could update this representation according to some driving input (Eliasmith 2007). The driving input may be a sensory signal carrying information about the current HD or location of an animal. Alternatively, the input may be an internal idiothetic signal representing some form of bodily self-motion.

Different kinds of idiothetic signal are found in particular areas of the rat brain. For example, the rat HD system is known to receive AHV signals representing the direction and speed of head rotation (Bassett and Taube 2001, 2005; Bassett et al. 2007). The confluence of HD and AHV signals within the rat HD system results in the development of conjunction cells representing combinations of HD and AHV (Chen et al. 1994; Stackman and Taube 1998; Sharp, Blair, et al. 2001a; Sharp, Tinkelman, et al. 2001b; Jercog et al. 2019). While the hippocampus, where place cells are found, appears to receive both forward motion signals and HD signals. The confluence of place, forward motion and HD signals within the hippocampus results in the development of conjunction cells representing combinations of place, forward motion and HD (Leutgeb et al. 2000; Sargolini 2006; Lu and Bilkey 2009; Chen et al. 2012). The CANN has to integrate such velocity signals in order to update its internal memory state correctly—that is, path integration.

A key issue is to understand how the synaptic connectivity in CANN models of HD cells and place cells could be set up through some form of biologically plausible learning process as the animal explores its sensory environment in the light. In particular, all of the synaptic connections within the network should self-organize using some form of biologically plausible “local” learning rule, in which the modification of the synaptic weights depends on locally available biological quantities such as the firing rates of the presynaptic and postsynaptic neurons.

During visually guided training, the synaptic connectivity within the CANN should self-organize to achieve the following three effects:

1. Develop appropriately structured synaptic connectivity from the visual input cells that endows the spatial cells with the observed firing properties of either HD cells or place cells in the light. This is a fundamental problem. The models need to show how HD cells and place cells may develop such different firing properties even though they receive common visual input signals from the sensory training environment.
2. Form conjunction cells that represent particular combinations of state and self-motion. Such conjunction cells are found in the rat HD system and the rat hippocampus where place cells are also present. In our past CANN models, these conjunction cells have played a key role in how the system learns to perform path integration (Walters and Stringer 2010; Walters et al. 2013; Page et al. 2018).
3. Form appropriately structured synaptic connectivity that permits the spatial cells to update their firing correctly as the animal moves in the dark—that is, path integration. To do this, the models need to learn to associate particular conjunctions of state and self-motion with specific changes in the state representation.

**Figure 1:**
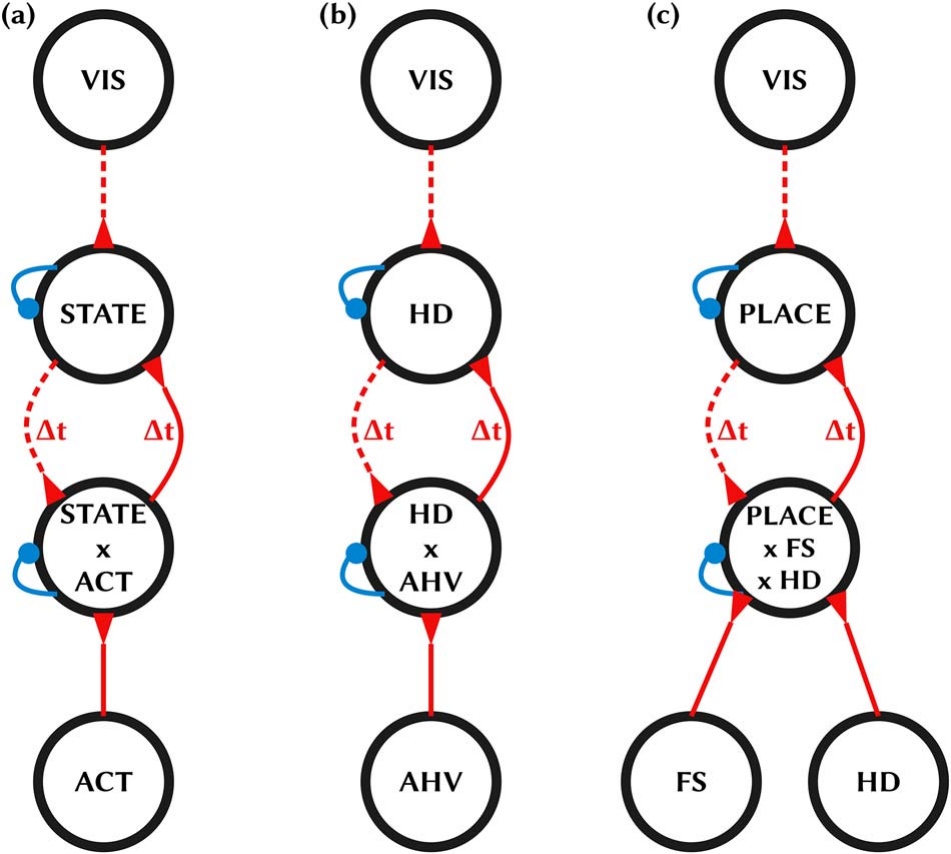
CANN architectures. Black circles represent sets of model neurons. Dashed lines represent sparse connectivity; solid lines represent full connectivity. Red indicates excitatory; blue inhibitory. Delayed connections are indicated with $\Delta t$: these are required for the network to be able to learn to perform path integration. Note that HD (*b*) and place (*c*) models are instantiations of the same abstract model (*a*). (*a*) General form of the architecture. Visual input cells, denoted VIS, encode the egocentric bearing to proximal and distal visual landmarks within the simulated sensory environment. The VIS cells send projections to the state cells, denoted STATE. The network also receives self-motion (idiothetic) signals from a layer of action cells, denoted ACT. STATE and ACT cells each send projections to a layer of combination cells, denoted STATE × ACT, which learn to represent particular combinations of state and action. Lateral inhibition in the STATE and STATE × ACT layers induces competitive learning, and thus the emergence of selectivity. (*b*) HD model. In order for the STATE cells to develop as HD cells, the ACT cells need to encode AHV. This, in turn, drives the combination cells to learn to represent combinations of HD × AHV. (*c*) Place model. In order for the STATE cells to develop as place cells, the ACT cells must encode velocity within the plane, which can be decomposed into the product FS × HD of FS and heading (via HD): in the model, this entails composing ACT from two corresponding subpopulations of cells. This, in turn, drives the combination cells to learn to represent combinations of PLACE × FS × HD.

In this paper, we introduce a fully self-organizing two-layer CANN model that achieves the above goals, including showing how either HD cells or place cells may develop depending on the kind of idiothetic signal available. The general CANN model is illustrated in Figure 1*a*. The network architecture consists of a layer of state cells that self-organize to become either HD cells or place cells, and a layer of conjunction cells that learn to represent combinations of the state and idiothetic signals. The state cells receive visual inputs from the sensory environment, as well as inputs from the conjunction cells. While the conjunction cells receive idiothetic inputs, as well as inputs from the state cells. In some simulations, the idiothetic inputs represent AHV, which leads to the state cells developing into HD cells. In other simulations, the idiothetic inputs represent combinations of forward velocity and HD, in which case the state cells develop into place cells. The bidirectional synaptic connections between the state cells and conjunction cells have axonal delays of a few milliseconds. This is essential to enabling the CANN models to learn to perform path integration (Walters et al. 2013; Page et al. 2018).

### The Development of State Cells into HD Cells or Place Cells

The population of state cells in our models, illustrated in Figure 1, operates as a competitive neural network (Rolls and Treves 1998). These kinds of networks self-organize their afferent synaptic connections using a biologically plausible learning mechanism, known as competitive learning, which is a thought to operate commonly in the sensory processing areas of the brain. The population of state cells “competes” with each other to respond to particular combinations of inputs via inhibitory interactions between the state cells, which in the brain would be mediated by inhibitory interneurons. At the same time, the afferent synaptic connections to the state cells are modified by an associative learning rule that modifies each synaptic weight in a manner depending on the firing rates of the presynaptic input cells and postsynaptic state cells. Individual synaptic weights are strengthened if the firing rates of the pre- and post-synaptic cells are correlated—that is, “cells that fire together wire together.” The effect of this kind of competitive learning is that the state cells learn to respond to particular combinations of their inputs, with different state cells responding to different combinations of inputs.

During visually guided training, as the simulated rat explores its environment, the state cells receive visual inputs from the environment as well as inputs from the population of conjunction cells. Competitive learning operates on all of the afferent synaptic connections from these inputs to the state cells. We find that the state cells either develop into HD cells if the idiothetic inputs are AHV as present in the HD system of the rat brain, or develop into place cells if the idiothetic inputs are combinations of forward motion and HD as appear to be present in the rat hippocampus.

### The Development of Conjunction Cells Encoding Combinations of State and Self-Motion

Our model incorporates cells with “conjunctive” response properties that respond to a combination of state and idiothetic (self-motion) signal. These correspond to the experimentally established conjunctive velocity-modulated HD and place cells noted above.

The layer of conjunction cells shown in Figure 1 is also modeled as a competitive neural network. The CANN simulations presented below demonstrate the self-organization of conjunction cells that encode particular combinations of state and self-motion. In the HD cell simulations, in which the network receives AHV idiothetic input signals, the conjunction cells learn to encode combinations of HD and AHV. While in the place cell simulations, in which the network receives idiothetic signals representing combinations of forward motion and HD, the conjunction cells learn to encode combinations of place, forward motion and HD. In both kinds of simulations, the conjunction cells self-organize their firing responses using the same kind of biologically plausible competitive learning mechanism. This kind of learning encourages individual conjunction neurons to learn to respond to particular combinations of afferent state and idiothetic signals. Hence, the emergent firing properties of the conjunction cells depends on the nature of the spatial representation encoded by the state cells, for example, HD or place, which codevelop simultaneously within the network. As explained above, the nature of the state representation that develops depends, in turn, on the kind of idiothetic inputs available. There is thus a rich interplay between the sensory and idiothetic input signals within the intermediate layers of the network during visually guided training and self-organization of the synaptic connectivity. Nevertheless, a common CANN model architecture can be used for both HD cell and place cell simulations, except for incorporating different idiothetic signals.

### The Development of Path Integration

In the CANN architecture illustrated in Figure 1, the bidirectional connections between the state cells and the conjunction cells have axonal delays Δ*t* of the order of a few milliseconds. This is essential for the ability of the network to learn to perform path integration at approximately the correct speed (Walters et al. 2013; Page et al. 2015, 2018). The axonal delays provide a natural time interval over which the network can learn temporal associations representing state transitions given an idiothetic signal representing some form of self-motion.

For example, as the simulated rat moves within its environment during visually guided training, the network can learn to associate the combination of state and idiothetic signal at time *t* with the successor state at time *t +* Δ*t*. After training, the self-organized synaptic connectivity will ensure that the representation of a combined state and idiothetic signal at time *t* will stimulate the correct successor state at time *t +* Δ*t*. In this way, the network can learn to perform accurate path integration. The population of conjunction cells play a key role in this process, in that they individually represent specific combinations of state and idiothetic signal that can be temporally associated with successor states represented by the population of state cells *Δt* later.

To elucidate further, consider an HD cell at time *t +* Δ*t*, receiving projections from visual inputs as well as conjunctive inputs. Due to the axonal propagation delays, the conjunctive neurons encode the HD and idiothetic signals at an earlier time *t*. Hebbian learning with an axonal delay Δ*t* incorporated entails that the HD cell learns to associate the representation of the current visual state with the earlier combination of HD and idiothetic (self-motion) signal represented by the conjunction cells. In the absence of driving sensory input, the recurrent connectivity between the state cells and conjunction cells therefore learns the temporal structure of state transitions: that is, the system learns to perform path integration. Moreover, this model explains precisely the functional importance of widely observed conjunctive representations.

The operation of these temporal association learning mechanisms within a two-layer CANN model of HD cells was originally discussed in detail by Walters et al. (2013). The learning is achieved using biologically plausible competitive learning mechanisms as described above, but where the synaptic connections and corresponding learning rules incorporate axonal delays.

This process of learning to perform path integration can be seen more generally as a form of inference learning, whereby the model learns to infer the next state given inputs representing the current state and self-motion. Successful path integration—that is, successful inference in the state-space model—requires that this learning process extracts the underlying state transitions of the animal in its environment from the changing visual and idiothetic input signals.

### Biologically Plausible Learning Mechanisms for Self-Organization of Synaptic Connectivity Within the Network Model

However, relatively few modeling studies have addressed how the synaptic connectivity may self-organize throughout the network model. Most models in the literature either simply hard-wire the synaptic connectivity (Redish et al. 1996; Zhang 1996; Samsonovich and McNaughton 1997; Tsodyks 1999; Goodridge and Touretzky 2000; Seung et al. 2000; Xie et al. 2002; Boucheny et al. 2005; Conklin and Eliasmith 2005; Song and Wang 2005; Burak and Fiete 2009; Bush and Burgess 2014; Si et al. 2014) or self-organize only some of the synaptic connections (Skaggs et al. 1995; Seung 1996; Gerstner and Abbott 1997; Stringer, Rolls, et al. 2002a; Stringer, Trappenberg, et al. 2002b; Stringer and Rolls 2006; Rennó-Costa and Tort 2017; Page et al. 2018). Understanding how such synaptic connectivity may be set up in the brain through learning is therefore an open problem. Moreover, biology imposes detailed constraints on the mechanisms underlying such learning. The principal such constraint is that synaptic plasticity utilizes learning rules that depend only on information local to each synapse. This constraint is well known, but others are often overlooked in modeling studies. For instance, CANNs are typically implemented using recurrent excitatory connectivity between the state cells, whereas no such connectivity is known to exist in the rat HD system (Sharp, Blair, et al. 2001a), and simulations show that such recurrent connections may be functionally deleterious to the accuracy of path integration (Page et al. 2015). Alternatively, the electrochemical nature of neural computation entails that signal propagation is temporally extended: information transmission between cells is subject to delays. Some of these biological constraints, such as the existence of axonal propagation delays, may have been exploited by evolution for functionally beneficial purposes. It is therefore imperative to explore the functional role of these biological constraints.

Our model presented below is fully self-organizing in a biologically plausible manner: all functionally relevant connectivity is plastic and subject to local associative Hebbian learning. This is an advance on earlier models developed by us (Stringer, Rolls, et al. 2002a; Stringer, Trappenberg, et al. 2002b; Stringer and Rolls 2006; Walters and Stringer 2010), in which the functionality of state cells depended on the existence of precomputed hard-wired connectivity. By showing that such hard-wiring is unnecessary, we extend the biological plausibility of this class of CANN model. More importantly, we are able to show how the network model may develop either HD cells if the idiothetic inputs represent AHV, or place cells if the idiothetic inputs represent combinations of forward movement and HD. Demonstrating this key theoretical result depends on the new step introduced in this paper of permitting the synaptic connections from the visual cells to the state cells to self-organize along with the other kinds of synaptic connection within the network.

### The Model Simulations Presented in this Paper

The CANN model presented below demonstrates that a single computational mechanism can explain the development of both HD cells and place cells. We consider two conditions. In the first condition, the conjunctive cell population receives idiothetic input representing the AHV of the simulated rat. While in the second condition, the conjunctive cells receive idiothetic signals representing a combination of forward motion and HD, which together represent the rat’s velocity through the environment. More precisely, in the first condition, the idiothetic input represents the time derivative of the rat’s HD; while in the second condition, it represents the time derivative of the rat’s location within the environment. Such idiothetic signals have been observed in the brain (Sharp 1996; Sharp, Blair, et al. 2001a; Wiener and Taube 2005; McNaughton et al. 2006; Ye et al. 2018). In the first condition, our model develops HD cells; in the second condition, it develops place cells. In both conditions, the visual input signal from the environment implemented during the training phase in the simulations is identical. Moreover, we show that a sufficiently rich sensory environment is necessary for the successful emergence of each kind of spatial representation: HD cells require distal landmarks, whereas place cells require proximal ones. Mechanistically, the external sensory input representing the changing state of the simulated rat, where the state may be HD or place within the environment, must be consistent with the idiothetic input signals representing the self-motion of the animal. In the absence of external sensory input from landmarks (Knierim 2002) or internal self-motion signals (Held and Hein 1963; Stackman et al. 2002) to generate these correlations during visually-guided learning, the same spatial representations do not emerge. Consequently, we show that breaking this rule leads to pathological development.

## Materials and Methods

### Operating Principle and Network Overview

#### Operation of the Model in the Light—Development of State Representations of Head Direction or Place

The core of our neural network model consists of two populations of cells, which we label “$\mathrm{STATE}$” and “$\mathrm{STATE}\times \mathrm{ACT}$” (abbreviated as $\mathrm{S}$ and $\mathrm{SA}$). The $\mathrm{S}$ cells learn without supervision to represent the state of the agent, such as HD or place in which the agent is situated. Whereas the $\mathrm{SA}$ cells learn a conjunctive representation of state and action (self-motion). Figure 1*a* shows the basic network architecture and connectivity. The two populations are interconnected by bidirectional connections with axonal propagation delays. The $\mathrm{S}$ cells receive a visual input signal via excitatory projections from a population of visual cells, as well as excitatory projections from the $\mathrm{SA}$ layer. The SA cells receive excitatory projections from the S cells, as well as a self-motion (idiothetic) signal that we call “ACT” (abbreviated). The model is completely self-organizing in that all of the connections develop through visually guided training using biologically plausible “local” learning rules as the agent explores its sensory environment in the light.

The neural representations of STATE and STATE × ACT emerge through unsupervised competitive learning as the agent moves through its environment in the light during the initial training phase. The competitive learning is effected by modifying the afferent excitatory connections to cells in each of the two populations by local Hebbian plasticity with weight vector normalization, while the cells within each of the populations compete with each other through lateral inhibition. Through these competitive learning mechanisms, each neuron in the two populations learns to respond to a distinct pattern of input activity—as is typical of competitive neural networks (Rolls and Treves 1998). In the Introduction, it was hypothesized that the nature of the state representation that develops would depend on the kind of self-motion signals incorporated into the network. Specifically, we proposed that the STATE cells would learn to represent HD if the network incorporated AHV signals, or would learn to represent the place in which the agent was situated if the network incorporated self-motion signals representing a combination of forward speed (FS) andHD.

Furthermore, the axonal transmission delays in the bidirectional connections between the STATE cells and the STATE × ACT cells enable the network to learn to associate a combination of state and action at one time with the resulting state a short while later—that is, effectively learning a state transition matrix. This temporal association then enables the network to perform accurate path integration as the agent moves in the dark, which we discussnext.

#### Operation of the Model in the Dark—Stabilization of State Representation and Path Integration

**Figure 2:**
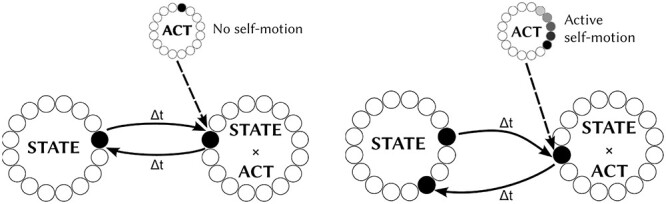
Illustration of the operating principles of the model in the absence of visual input when the agent is either stationary or moving. **Left panel** (a), stationary case, illustration of how the network is able to maintain a stable representation of the current state of the agent when the active cells within ACT layer represent no self-motion. Black and white circles represent active and inactive cells respectively; arrows denote connectivity. The active “no self-motion” cells within the ACT layer stimulate the subpopulation of cells within the STATE × ACT layer representing a combination of no self-motion and the current state. Then the stable representation of STATE is maintained by symmetric bidirectional connections between the STATE and STATE × ACT layers set up during learning as the agent explores its sensory training environment in the light. **Right panel** (b), active case, illustration of how the network is able to update its state representation when there is an “active self-motion” signal represented by the ACT layer. In this case, the representation within the STATE layer is continuously updated by the asymmetric bidirectional connections between the STATE and STATE × ACT layers set up during learning. That is, given some action, the bidirectional connectivity with axonal transmission delays effectively acts as a state transition matrix, by learning how states are caused by earlier state-action combinations. This capability is known as path integration. The learning rules used to adjust the strengths of the bidirectional connections during training ensure that the resulting asymmetry in these connections is appropriate to update the state at the correct velocity for the current self-motion signal.

After the model has been trained by allowing the simulated agent to explore its sensory training environment in the light, the model is able to maintain and update its state representation in the dark using self-motion (idiothetic) signals. This property of HD cells and place cells in the brain is known as path integration. For example, when the model incorporates AHV self-motion signals, HD cells develop during training in the light. In this case, after training, the AHV signals are able to update the representation of HD in the dark. Similarly, when the network incorporates self-motion signals representing combinations of FS and HD, then after training these self-motion signals are able to update the representation of place in the dark. How the model achieves this is illustrated in Figure 2.

The network shown on the left of Figure 2 illustrates the case when there is no self-motion signal represented by the ACT layer in the dark. Specifically, there is a subpopulation of cells in the ACT layer that explicitly represent no self-motion. After training, these ACT cells representing no self-motion stimulate the subpopulation of combination cells in the STATE × ACT layer that have learned to represent combinations of a particular state and no self-motion. During training in the light, the STATE cells and the subpopulation of STATE × ACT cells representing no self-motion develop “symmetric” bidirectional connections between themselves. This is because the agent simply remains in a fixed state during periods of no self-motion as the agent is trained. In this situation, the symmetric bidirectional connections between the STATE cells and STATE × ACT cells representing no self-motion that have developed during training are able to stabilize the representation of the agent’s state in the dark, where the state may be either HD or place.

In contrast, the network shown on the right of Figure 2 illustrates the case where there is an active self-motion signal in the dark. Specifically, there is a subpopulation of cells in the ACT layer that explicitly represent some form of active self-motion such as AHV or combination of FS and HD. After training, these ACT cells representing active self-motion stimulate the subpopulation of combination cells in the STATE × ACT layer that have learned to represent combinations of a particular state and that form of active self-motion. During training in the light, the STATE cells and the subpopulation of STATE × ACT cells representing active self-motion develop “asymmetric” bidirectional connections between themselves. This is because the agent is continuously changing its state in accordance with the currently active self-motion signals as the agent is trained. In this situation, the asymmetry in the bidirectional connections is able to continuously update the state representation at the correct velocity so as to track the true state of the agent in the dark. In particular, the learning rules used to modify the strengths of the bidirectional connections during training in the light are able to structure the asymmetry in these bidirectional connections such that path integration is performed accurately by the network during active self-motion in thedark.

Effectively, the bidirectional connections between the STATE cells and STATE × ACT cells, which incorporate axonal transmission delays, are able to learn a state transmission matrix encoding how either no self-motion or different kinds of active self-motion lead to changes in state. The form of self-motion represented by the ACT cells stimulates the corresponding subpopulation of STATE × ACT cells that represent that particular form of self-motion. This, in turn, activates the appropriate subset of bidirectional connections between the STATE and STATE × ACT layers, ensuring that the state is either held static for no self-motion or continuously updated according to the current form of active self-motion. Thus, the self-motion signal is effectively able to activate the appropriate subset of bidirectional connections to ensure that the state representation is updated correctly for the currently active self-motion signal. The above operational principles of the network model during training in the light and testing in the dark have been previously explained and demonstrated by Walters et al. (2013) and Page et al. (2018).

### Neural Dynamics

All of the cells in our neural network models are rate-coded. That is, we do not explicitly simulate the timings of action potentials emitted by neurons. Instead, our models represent the time averaged firing rates of cells, where each firing rate is bounded within the interval [0,1].

Our models simulate the dynamical behavior of the STATE cells (S) and STATE × ACT cells (SA) using coupled differential equations governing the evolution of their activities through time. While the activities of the visual input cells (VIS) and self-motion input cells (ACT) are imposed through time according to the programmed behavior of the simulated agent as it explores its sensory training environment.

The general form of rate-coded dynamics governing the behavior of the S and SA cells is as follows.

For each $\mathrm{S}$ and $\mathrm{SA}$ cell $i$, the instantaneous postsynaptic activation ${h}_i$ is given by

(1) \begin{equation*} {h}_i(t)=\sum_M{\phi}^M\sum_j{W}_{ij}^M{r}_j^M\left(t-\Delta t\right) \end{equation*}

The first summation in the above equation is over the presynaptic layers of cells, denoted by the index *M*, which send connections to the postsynaptic S or SA cell *i*. This summation is expanded below for S and SA cells in connectivity between layers section. The parameter ${\phi}^M$ is a so-called “scaling constant” (Scaling Constants section). ${W}_{ij}^M$ is the synaptic weight from presynaptic cell *j* in layer *M* to the postsynaptic *S* or *SA* cell *i*. The term ${r}_j^M(t-\Delta t)$ is the firing rate of the presynaptic cell *j* in layer *M* at time $(t-\Delta t)$ where $\Delta t$ is an axonal transmission delay. In the bidirectional connections between the S and SA cells, $\Delta t$ is drawn from a uniform distribution over the biologically realistic interval between 10 and 30 ms, in order to incorporate a nonzero axonal transmission delay. Such transmission delays are needed to enable the model to learn to associate combinations of states and actions with the states that result a short time later. After training, the model is able to use these learned temporal associations to perform path integration in the dark. However, $\Delta t$ is set to zero for the input connections from the visual cells (VIS) and the self-motion cells (ACT), since these connections do not need to learn such temporal associations needed for path integration.

The firing rate ${r}_i(t)$ of each $\mathrm{S}$ and $\mathrm{SA}$ cell $i$ is given by integrating the following ordinary differential equation

$$ {\tau}_i\frac{\mathrm{d}{r}_i}{\mathrm{d}t}=-{r}_i+\frac{1}{1+{e}^{-2{\beta}_i\left({h}_i(t)-{\alpha}_i\right)}} $$

where ${\tau}_i$ is the time constant, ${\beta}_i$ gives the slope for the logistic transfer function, ${\alpha}_i$ gives the firing threshold, and ${h}_i$ is the postsynaptic activation of cell *i* defined above. Typical values for these parameters are $\{{\tau}_i={10}^{-2};{\alpha}_i=2.0;{\beta}_i=1.0\}$; see Table 1.

**Table 1:** Model parameters used in simulation studies 1–5

	Study 1 (HD)	Studies 2, 3, 5 (HD)	Studies 4,5 (place)
**Number of cells *N* in each neuronal layer**			
VIS, per landmark	200	100	100
STATE	1000	1000	1000
STATE × ACT	2000	2000	2000
AHV, per action state (i.e., clockwise rotation, anticlockwise rotation, or no rotation)	1	1	–
FS, per action state (i.e., forward motion or no forward motion)	–	–	1
HD	–	–	200
**Neuronal time constants τ**			
STATE	0.01	0.01	0.01
STATE × ACT	0.01	0.01	0.01
**Threshold α, slope β in logistic transfer function**			
STATE	2.0, 1.0	2.0, 1.0	2.0, 1.0
STATE × ACT	2.0, 1.0	2.0, 1.0	2.0, 1.0
**Width σ of Gaussian activity profiles of VIS neurons and HD idiothetic (ACT) neurons (radians)**			
VIS	π/9	π/9	π/9
HD idiothetic	π/9	π/9	π/9
**Parameter ϕ scaling strengths of connections between neuronal layers**			
VIS → STATE	30.0	36.0	36.0
STATE × ACT → STATE	22.0	27.0	27.0
STATE → STATE × ACT	12.0	9.0	9.0
AHV → STATE × ACT	50.0	50.0	–
FS → STATE × ACT	–	–	25.0
HD → STATE × ACT	–	–	6.25
**Scaling parameter** $\overline{\phi}$ **for inhibitory connections**			
VIS → STATE	0.045	0.036	0.036
STATE → STATE	0.27	0.8	0.8
STATE × ACT → STATE × ACT	2.0	1.2	1.2
**Training parameters**			
Learning rate *k*	0.02	0.02	0.02
Training time (s)	1200	3600	3600

Notes: The VIS → STATE and STATE → STATE × ACT connections were sparse (i.e., diluted) with a sparsity of 5%. That is, each neuron in the postsynaptic (receiving) layer received afferent connections from only 5% of the neurons in the presynaptic (sending) layer. The bidirectional STATE → STATE × ACT and STATE × ACT → STATE axonal connections had transmission delays Δt randomly selected from a uniform distribution between 10 and 30 ms. No other types of connection incorporated delays. The differential equations were solved numerically using a forward Euler time-stepping scheme with a timestep of 2^−10^ s (~1 ms).

The equations are integrated with the forward-Euler algorithm and a timestep of ${2}^{-10}$ s (~1 ms).

### Scaling Constants

The scaling constants ${\phi}^M$ allow the relative input strengths of afferent signals from different layers to be individually adjusted. This is necessary because all of the cell firing rates within the network are constrained by the dynamics to lie within the range [0,1]. For the simulations presented below, these parameters were found by manual experimentation (see parameter Table 1).

### Connectivity Between Layers

As shown in Figure 1*a*, the STATE cells $\mathrm{S}$ receive visual inputs (denoted VIS) as well as projections from the STATE × ACT cells $\mathrm{SA}$. The VIS connections are given a relatively high scaling constant ${\phi}^M$ (see parameter Table 1), to force the visual signal to drive the network during training. In rats, the visual signal anchors the spatial representation and thus overrides the idiothetic signal. Both sets of afferent connections to the STATE cells are plastic (Learning Rules section). As described above, the SA$\to$S projections have axonal transmission delays $\Delta t$ of 10 ms, while the VIS$\to$S projections have zero delays. Also, the VIS$\to$S connections are sparsely connected with a sparsity of 5%, while the SA$\to$S projections are fully connected.

Sparse connectivity is implemented in certain types of connection within the network models in order to prevent the emergence of “continuous transformation” (CT) *learning*. CT learning is an invariance learning mechanism that has previously been used to model the emergence of transform invariant visual representations in the brain (Stringer and Rolls 2006). Its effect is to encourage a small subset of cells in a competitive layer to respond invariantly to all of the smoothly varying input patterns. This kind of learning would destroy the state specific representations that we are seeking to develop within the HD cell and place cell models presented here. However, it has previously been found that CT learning can be eliminated in such network models by implementing sparse connectivity in certain types of connection (Walters et al. 2013; Page et al. 2018). We adopt the same approachhere.

Expanding the activation equation (1) in the case of the STATE cells, we therefore have

(2) \begin{equation*} {\displaystyle \begin{array}{c}{h}_i^S(t)={\phi}^{\mathrm{VIS}}\sum_j{W}_{ij}^{\mathrm{VIS}}{r}_j^{\mathrm{VIS}}(t)-\overline{\phi^{\mathrm{VIS}}}{\chi}^{\mathrm{VIS}}(t)+{\phi}^{\mathrm{S}\mathrm{A}}\sum_j{W}_{ij}^{\mathrm{S}\mathrm{A}}{r}_j^{\mathrm{S}\mathrm{A}}\left(t-\varDelta t\right)\\ -\overline{\phi^{\mathrm{S}}}\sum_j{r}_j^{\mathrm{S}}(t)\end{array}} \end{equation*}

where $\overline{\phi^M}$ denotes the scaling constants for inhibitory connections (Connectivity between Layers section), and ${\chi}^{\mathrm{VIS}}(t)=1$ when visual inputs are active, and 0 otherwise. The term ${\chi}^{\mathrm{VIS}}(t)$ effectively provides a form of feedforward inhibition from the visual layer to the STATE cells, which counterbalances the excitatory input from the visual cells when they are active in the light. The last term of the above equation implements mutual inhibition and competition between the STATE cells, which is needed for competitive learning to be able to operate in the afferent connections to this layer.

The STATE × ACT cells $\mathrm{SA}$ receive self-motion inputs (denoted ACT) as well as projections from the STATE cells $\mathrm{S}$. Both sets of afferent connections to the STATE × ACT cells are plastic (Learning Rules section). The S$\to$SA projections have axonal transmission delays $\Delta t$ drawn from a uniform distribution over the interval between 10 and 30 ms, while the ACT$\to$SA projections have zero delays. Also, the ACT$\to$SA connections are fully connected, while the S$\to$SA projections are sparsely connected with a sparsity of 5%.

In the model simulations presented below, the ACT cells may represent either AHV or combinations of FS andHD.

In the case of the ACT cells representing AHV, expanding equation (1) or the STATE$\times$ACT cells, we have

(3) \begin{equation*} {\displaystyle \begin{array}{c}{h}_i^{\mathrm{S}\mathrm{A}}(t)={\phi}^{\mathrm{AHV}}\sum_j{W}_{ij}^{\mathrm{ACT}}{r}_j^{\mathrm{AHV}}(t)+{\phi}^{\mathrm{S}}\sum_j{W}_{ij}^{\mathrm{S}}{r}_j^{\mathrm{S}}\left(t-\varDelta t\right)\\ -\overline{\phi^{\mathrm{S}\mathrm{A}}}\sum_j{r}_j^{\mathrm{S}\mathrm{A}}(t)\end{array}} \end{equation*}

where $\overline{\phi^{\mathrm{SA}}}$ denotes the scaling constant for the inhibitory connections between the SA cells (Inhibitory Interactions within and between Layers section). The last term of the above equation implements mutual inhibition and competition between the STATE × ACT cells, which is again needed for competitive learning to operate in the afferent connections to this layer.

For the case of ACT cells representing combinations of FS and HD, expanding equation (1) for the STATE$\times$ACT cells, we have

(4) \begin{equation*} {\displaystyle \begin{array}{c}{h}_i^{\mathrm{S}\mathrm{A}}(t)={\phi}^{\mathrm{FS}}\sum_j{W}_{ij}^{\mathrm{FS}}{r}_j^{\mathrm{FS}}(t)+{\phi}^{\mathrm{HD}}\sum_j{W}_{ij}^{\mathrm{HD}}{r}_j^{\mathrm{HD}}(t)+{\phi}^{\mathrm{S}}\sum_j{W}_{ij}^{\mathrm{S}}{r}_j^{\mathrm{S}}\left(t-\varDelta t\right)\\ -\overline{\phi^{\mathrm{S}\mathrm{A}}}\sum_j{r}_j^{\mathrm{S}\mathrm{A}}(t).\end{array}} \end{equation*}

### Inhibitory Interactions Within and Between Layers

In order to promote competitive learning within both the layer of $\mathrm{S}$ cells and the layer of $\mathrm{SA}$ cells, each of these layers incorporates mutual inhibitory interactions between the cells. This ensures that cells within each layer compete with each other to learn to represent incoming activity patterns. In the brain, such inhibitory interactions could be effected through inhibitory interneurons. However, our network models do not explicitly incorporate additional populations of inhibitory cells, which would be computationally expensive. Instead, we add direct inhibitory connections between the cells in both the S and SA layers, with the scaling constant $\overline{\phi}$ chosen as described in Scaling Constants section (see parameter Table 1). This leads to the term

$$ -\overline{\phi^M}\sum_j{r}_j^M(t) $$

in the activation equations (2)–(4).

The visual input from the VIS layer to the STATE layer needs to be strong enough to drive the network model during training in the light. However, the network must also be capable of maintaining and updating its state representation in the dark, when it is driven by the self-motion inputs from the ACT layer. Consequently, we have found in past modeling studies that the strong excitatory visual input drive needs to be balanced by additional feedforward inhibition from the VIS layer to the STATE layer. The feedforward inhibition is active whenever the excitatory visual signals are present in the light. It is modeled by incorporating the following term into equation (2) governing the activation of the STATE cells:

$$ -\overline{\phi^{\mathrm{VIS}}}{\chi}^{\mathrm{VIS}}(t) $$

where $\overline{\phi^{\mathrm{VIS}}}$ is a scaling constant, and ${\chi}^{\mathrm{VIS}}(t)=1$ when the visual input is active and 0 otherwise.

The inhibitory connections described above are fixed and not subject to plasticity during training of the network models.

### Learning Rules

The excitatory synaptic connection weights throughout the network are subject to a biologically plausible form of Hebbian plasticity

$$ \frac{\mathrm{d}{W}_{ij}}{\mathrm{d}t}=k{r}_i(t){r}_j\left(t-{\left(\varDelta t\right)}_{ij}\right) $$

where ${W}_{ij}$ is the strength of the connection from presynaptic cell *j* to postsynaptic cell *i*, ${r}_i$ and ${r}_j$ are respectively the firing rates of cells *i* and *j*, ${(\varDelta t)}_{ij}$ is the axonal transmission delay associated to the connection, and *k* is the learning rate where a typical value is ${10}^{-2}$ (see parameter Table 1).

In the bidirectional connections between the S and SA cells, ${(\Delta t)}_{ij}$ is set to a nonzero value drawn from the uniform distribution over the closed interval from 10 to 30 ms. These transmission delays enable the model to learn to associate combinations of states and actions with successor states. After training in the light, such temporal associations enable the network to perform path integration in the dark. However, ${(\Delta t)}_{ij}$ is set to zero for the input connections from the VIS cells to the STATE cells and from the ACT cells to the STATE × ACT cells. This is because these kinds of connections do not need to learn the temporal/causal associations needed for the network to be able to perform path integration.

The sparsity structures of the synaptic weight matrices $W$ are maintained during learning; that is, no new synapses are formed.

For clarity, the learning rules used to modify the four kinds of excitatory connection within the network models are as follows.

The connections from the VIS layer to the STATE layer are modified according to:

$$ \frac{\mathrm{d}{W}_{ij}^{\mathrm{VIS}}}{\mathrm{d}t}=k{r}_i^S(t){r}_j^{\mathrm{VIS}}(t) $$

The connections from the STATE × ACTION layer to the STATE layer are modified according to:

$$ \frac{\mathrm{d}{W}_{ij}^{\mathrm{S}\mathrm{A}}}{\mathrm{d}t}=k{r}_i^{\mathrm{S}}(t){r}_j^{\mathrm{S}\mathrm{A}}\left(t-{\left(\varDelta t\right)}_{ij}^{\mathrm{S}\mathrm{A}}\right) $$

The connections from the STATE layer to the STATE × ACTION layer are modified according to:

$$ \frac{\mathrm{d}{W}_{ij}^{\mathrm{S}}}{\mathrm{d}t}=k{r}_i^{\mathrm{S}\mathrm{A}}(t){r}_j^{\mathrm{S}}\left(t-{\left(\varDelta t\right)}_{ij}^{\mathrm{S}}\right) $$

The connections from the ACT layer to the STATE × ACTION layer are modified according to:

$$ \frac{\mathrm{d}{W}_{ij}^{\mathrm{ACT}}}{\mathrm{d}t}=k{r}_i^{\mathrm{SA}}(t){r}_j^{\mathrm{ACT}}(t) $$

In order to prevent the afferent synaptic weights of any S or SA cell *i* growing too large, the weight vector of each such cell is renormalized after each timestep during training so that

$$ \sqrt{\sum_j{W_{ij}}^2}=1. $$

where the sum is over all the presynaptic cells *j*. Such a renormalization process may be achieved in biological systems through synaptic weight decay (Oja 1982; Rolls and Treves 1998). The renormalization helps to ensure that the learning rules are convergent in the sense that they settle down over time to steady values, that is, the weights do not grow unbounded.

### Simulation Protocol and Visual Inputs

**Figure 3:**
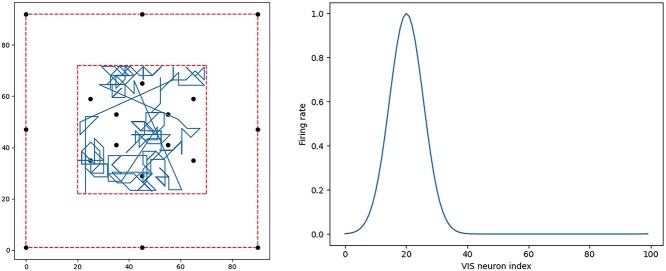
Visual training environment and visual inputs to the models. **Left:** The visual training environment contains a mixture of landmarks, indicated by black circles, which may be either proximal (nearby) or distal (far away). Distal landmarks may be placed either at infinite distance or, as in the more ecologically valid configuration shown here, on the boundary of the finite environment. The red dashed lines represent transparent (invisible) barriers, which delineate the extent of both the entire environment (outer barrier, with visible distal landmarks) and the navigable portion (inner barrier). An example of a typical random walk undertaken by the agent is shown. The units on the axes are arbitrary units of distance. **Right**: The models receive visual input signals from separate subpopulations of VIS cells, each subpopulation encoding the egocentric bearing to a specific landmark in Gaussian-tuned firing. The figure exemplifies the visual input at a single point in time from one subpopulation representing the bearing to a single landmark. The VIS cell indices are shown on the abscissa, sorted to make the Gaussian tuning explicit. In the example environment shown (left), with 18 landmarks, the model has a total of 1800 VIS neurons with the bearing to each landmark encoded by 100 neurons.

The simulated agent is trained and tested within a two-dimensional square visual training environment that contains a mixture of proximal (nearby) and distal (far away) landmarks. The environment is shown in Figure 3 (left). The black circles represent the proximal landmarks, which the agent can move around. The distal landmarks are modeled as lying at infinity, and so are not shown.

In each experiment, the simulation protocol is similar. During the initial training phase of the simulation, the agent explores the visual environment, usually according to a random walk as shown in Figure 3 (left). During this phase, activity within the network is driven by both the visual inputs (VIS layer) representing the proximal and distal landmarks as well as the self-motion signals (ACT layer). At the same time, the plastic synaptic weights are modified according to the learning rules described above.

The visual inputs encoded by the VIS layer represent the egocentric bearings from the simulated agent to the proximal and distal landmarks. Each landmark (proximal or distal) is associated with a subpopulation of VIS cells, with each subpopulation having the same size *M* and encoding the bearing of the agent to the corresponding landmark via a Gaussian population code, as follows. Within a given subpopulation, each cell *j* has a Gaussian response profile tuned to respond maximally to a particular bearing to the corresponding landmark, with these preferred bearings ${\theta}_j^{\mathrm{pref}}$ being uniformly distributed around the circular interval [0,360°]. Let ${\theta}_i$ denote the agent’s bearing to landmark *i*. Let ${\mathrm{s}}_j=\min (|{\theta}_j^{\mathrm{pref}}-{\theta}_i|,2\pi -|{\theta}_j^{\mathrm{pref}}-{\theta}_i|)$ be the difference between the preferred and actual bearings. Then, the VIS cell *j* responds with a firing rate given by

(5) \begin{equation*} {r}_j^{\mathrm{VIS}}=\exp \left[\frac{-{s}_j^2}{2{\left({\sigma}^{\mathrm{VIS}}\right)}^2}\right] \end{equation*}

where ${\sigma}^{\mathrm{VIS}}$ is the width of the Gaussian. In this way, the egocentric bearing to a proximal landmark is represented by a bump of activity within the corresponding ring of VIS cells, as shown in Figure 3 (right).

Within a simulation, there can be arbitrarily many proximal or distal landmarks. If ${L}^{\mathrm{prox}}$is the number of proximal landmarks and ${L}^{\mathrm{dist}}$ the number of distal landmarks, then the total number of VIS cells will be ${N}^{\mathrm{VIS}}=M\cdotp ({L}^{\mathrm{prox}}+{L}^{\mathrm{dist}})$, where *M* is the number of VIS neurons encoding each landmark.

The firing rate computation described above is agnostic to the type of landmark (i.e., proximal vs. distal), but to simplify the simulation code we specialize the computation of the agent’s bearing ${\theta}_i$ to landmark *i* according to the type of landmark as follows.

Supposing *i* is a proximal landmark (or distal landmark at finite distance), then it is associated with a position vector ${q}_i$. The vector from the agent’s current position $p$ to the landmark is therefore given by ${v}_i={q}_i-p$. The egocentric bearing is then computed as ${\theta}_i=\arctan ({v}_i^{(y)}/{v}_i^{(x)})-{\theta}_{HD}$, where ${v}_i^{(x)}$ and ${v}_i^{(y)}$ are respectively the *x* and *y* components of the vector ${v}_i$, and ${\theta}_{\mathrm{HD}}$ is the agent’s current heading.

Alternatively, a distal landmark *i* at infinite distance has no position, only an angle ${\omega}_i$ around the horizon. Consequently, the agent’s egocentric bearing to the distal landmark *i* is simply given by ${\theta}_i={\omega}_i-{\theta}_{\mathrm{HD}}$, where ${\theta}_{\mathrm{HD}}$ is again the agent’s current heading.

The visual representation of the egocentric bearings to the distal landmarks is, of course, perfectly correlated with the HD of the agent. It is for this reason that the simulations reported below find that the presence of the distal cues is important for the development of HD cells within the network model during training. However, it is important to note that we never directly impose HD cell like responses on the STATE cells during training and testing, as was done in earlier modeling studies (Walters et al. 2013; Page et al. 2018). Instead, the strengths of the excitatory synaptic connections from the VIS cells to the STATE cells are randomized at the start of the simulation, which initially endows the STATE cells with unstructured firing responses. These synaptic connections then self-organize by competitive learning using the learning rules described above during visually guided training as the agent explores its environment in the light. It is this training process that ultimately endows the STATE cells with their learned firing characteristics, which may come to represent either the agent’s HD or place within the environment.

### Idiothetic Inputs

The self-motion (idiothetic) signals are represented by the layer of ACT cells. The kind of self-motion signals incorporated into the network model is changed between simulations. In particular, we investigate how alternative kinds of self-motion signal lead to the development of different kinds of spatial representations in the network. In simulations showing the emergence of HD cells, the ACT cells represent AHV. While in simulations showing the emergence of place cells, the ACT cells represent combinations of FS andHD.

### AHV Signal

In simulations demonstrating the emergence of HD cells, the network incorporates AHV signals. We model the AHV input using three ACT cells. One cell is on (i.e., has firing rate 1.0) when the agent is not rotating; a second cell is on when the agent rotates clockwise; and a third cell is on when the agent rotates anticlockwise. When one cell is on, the other cells are off (i.e., have firing rate 0.0). In this paper, we only model these three states of self-motion. Transitions between the states are sharp (i.e., discontinuous step functions), so there is no simulation of continuous acceleration or deceleration, and no corresponding smooth changes in the AHV signal.

### FS and HD Signals

In simulations demonstrating the emergence of place cells, the network incorporates a combination of FS and HD signals. The FS and HD signals are represented by two distinct subpopulations of ACT cells.

The FS signal is implemented in a rather similar manner to the AHV signal. We only simulate two FS states, each of which is represented by a separate ACT cell. The first FS state is no forward motion, with its corresponding ACT cell active (firing rate 1.0). The second FS state is forward motion with unit speed, with the other ACT cell active. Transitions between these two FS states are sharp, and exactly one of the two ACT cells encoding these FS states is active at anytime.

For the HD signal, we do not use the HD representation learned by the network model with AHV self-motion inputs, though this will be pursued in a future study. Instead, we create a ring of ACT cells that mimic the firing characteristics of HD cells found in the brain. Each ACT cell in the ring responds maximally when the simulated agent is oriented towards a preferred HD, with a Gaussian response field centered around the preferred HD of the cell. Moreover, each ACT cell in the ring is tuned to a different preferred HD, and these cells are distributed evenly around the circular HD interval [0, 360°]. Essentially, this HD representation is somewhat similar to the visual representation of a distal landmark described above.

### Testing Protocols

After a training period of fixed length (see parameter Table 1), synaptic plasticity is disabled. Testing the network is then carried out with learning switchedoff.

The learned response characteristics of the STATE cells within the network are tested using a number of different protocols as follows.

First, in studies 1 through 3, we measure the tuning of each cell in the STATE population by recording the cell’s response as the agent, still receiving visual input, is rotated through a full turn in each direction. We then average the two sets of responses per angle per cell. In study 3, to test path integration, we switch off the visual input (and feedforward visual inhibition) halfway through thetest.

In studies 2, 4, and 5, we divide the environment into a Cartesian (square) lattice of testing points, and move the agent systematically across this lattice. At each lattice point, the agent makes a full rotation in each direction, according to each available AHV, that is, each clockwise and anticlockwise rotation. We record the neural activity of each model during this process, and compute the average response of each cell to each lattice point (for place tuning) and HD (for HD tuning).

### Decoding the HD Signal in Path Integration Studies

In studies of path integration, we need to decode the HD signal represented by the STATE cells as the agent rotates in the dark. This is necessary to assess the ability of the network to accurately track the changing HD of the simulated agent by integrating the AHV signal in the absence of visual input. In order to mimic approximately how the brain might decode the HD signal, we learn a linear model after training during rotation in the light, and then use the learnt model to decode the signal from the STATE cells during rotation in the dark. More precisely, we use the Bayesian linear regression implementation provided by the Python scikit-learn package (Pedregosa et al. 2011) to learn the weights of a (Bayesian) least-squares regression model and later use these weights to infer the signal during path integration. That is, writing ${R}^{\mathrm{S}}$ for the ${N}^{\mathrm{S}}\times T$ matrix of firing rates over time (where $T$ is the number of sampled data points in the time series and ${N}^{\mathrm{S}}$ is the number of STATE cells), we assume that the HD time series can be decoded linearly by $\mathrm{HD}={R}^{\mathrm{S}}W+\epsilon$ (where $W$ is here a matrix of decoding weights, and $\epsilon$ is a normally distributed noise term), and learn the decoding weights $W$ that minimize the error ${\Vert \mathrm{HD}-{R}^{\mathrm{S}}W\Vert}_2^2$ using the BayesianRidge algorithm of scikit-learn. A detailed explanation of the algorithm is available in Bishop (2006), §3.3.

### Simulation Parameters

 

### Code Availability

All code required for performing and analyzing the simulations presented here is available online at https://github.com/OFTNAI/telos.

## Results

We present results from five simulation studies. These studies investigate the effects of varying two key model factors:

1. The nature of the self-motion (idiothetic) input to the model. This is varied between two alternative options: the model receives either AHV signals, or a combination of FS andHD.
2. The kinds of visual landmarks present within the environment. The visual landmarks may be proximal (nearby objects that the simulated agent can move amongst), or distal (far away), or a mixture of both proximal and distal. The agent’s visual input neurons represent the egocentric bearings to these landmarks.

The simulations described below demonstrate the following important model behaviors. When the self-motion signals (the ACT cells in Fig. 1) represent AHV and the environment contains distal visual landmarks, then the STATE cells learn to represent the HD of the agent, replicating the firing properties of HD cells found in the rat brain. However, when the self-motion signals represent a combination of FS and HD, and the environment contains proximal visual landmarks, then the STATE cells learn to represent the location of the agent within the environment, replicating the response properties of place cells in the brain. These model behaviors are robust in that the simulations continue to develop HD cells or place cells, depending on the nature of the self-motion signals, even when there is a mixture of both proximal and distal landmarks. However, HD cells do not develop if the environment contains only proximal visual landmarks but no distal visual landmarks. Contrariwise, place cells do not develop if the environment contains only distal visual landmarks but not proximal visual landmarks. This implies that the HD cells become anchored predominantly to the distal visual landmarks, while place cells become associated with more proximal visual landmarks. Experimental evidence for this has been found in studies with rats (Yoganarasimha 2006; Renaudineau et al. 2007; Knierim and Hamilton 2011; Page and Jeffery 2018).

### AHV Idiothetic Input Generates an HD Representation

#### Study 1

First, we demonstrate that the model is able to learn a HD representation when receiving AHV self-motion input, in a simple environment containing five distal visual landmarks, situated at infinite distance, evenly spaced around the horizon, and never occluded. The neural network architecture simulated is that shown in Figure 1*b*.

The model is first trained with the agent rotating within the environment in the light. During training, all of the synaptic connection weights within the model are self-organized using the learning rules described in the Methods section.

Because these landmarks are modeled as if at infinite distance, forward motion has no effect on the bearing. So rather than simulate the agent performing a random walk through the environment, we hold the agent at the origin of the map and choose randomly whether and in which direction to rotate the agent. Furthermore, we do not simulate smooth angular acceleration. Instead the model jumps instantaneously between different angular head velocities. Each type of rotation corresponds to a distinct AHV representation, and transitions between AHV representations are sharp, corresponding to transitions between rotation states.

**Figure 4:**
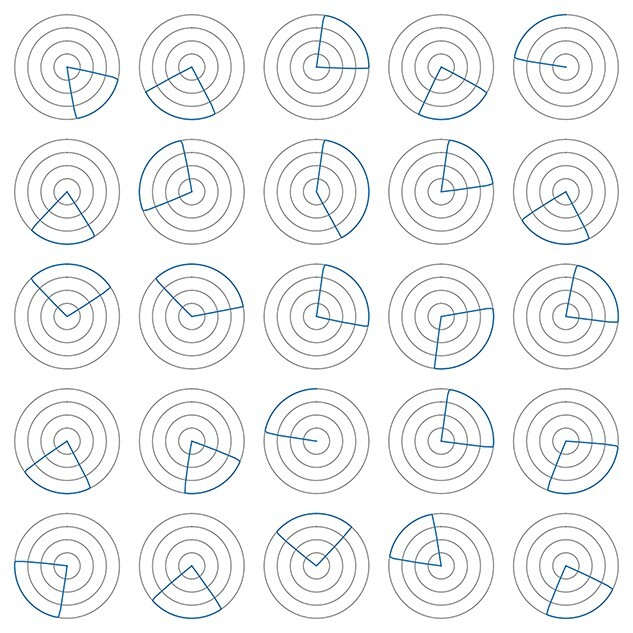
Simulation of the HD cell model shown in Figure 1*b* with AHV self-motion inputs. The model is first trained with the agent rotating within the environment in the light with five idealized distal visual landmarks present, situated on the horizon at infinite distance from the agent, spaced evenly around the circle. After training, the model is tested with the simulated agent rotating on the spot through 360° in the same environment in the light. The figure shows the directional tuning curves of 25 randomly selected STATE cells during testing, in the form of polar firing rate plots, with the gray circles representing 25%, 50%, 75%, and 100% of the maximum firing rate. We observe the characteristic directional selectivity of HD cells. That is, each STATE cell responds selectively to a localized region of the HD space, with different STATE cells responsive to different HDs. The preferred HDs of the STATE cells cover the entire circle. Note that the discontinuities in some of the subplots (e.g., top right) are artifacts of the sampling process used for data collection, occurring when the last data point of the sampled time series does not return to the starting value.

After training the model in this way, we measure the tuning of each cell in the STATE population by recording the cell’s response as the agent, still receiving visual input, is rotated through a full turn in each direction. We then average the two sets of responses per angle per cell. A random sample of these tunings is plotted in polar form in Figure 4: distance from the origin denotes the neuronal firing rate. We observe the directional selectivity characteristic of HD cells, distributed around the full circle.

**Figure 5:**
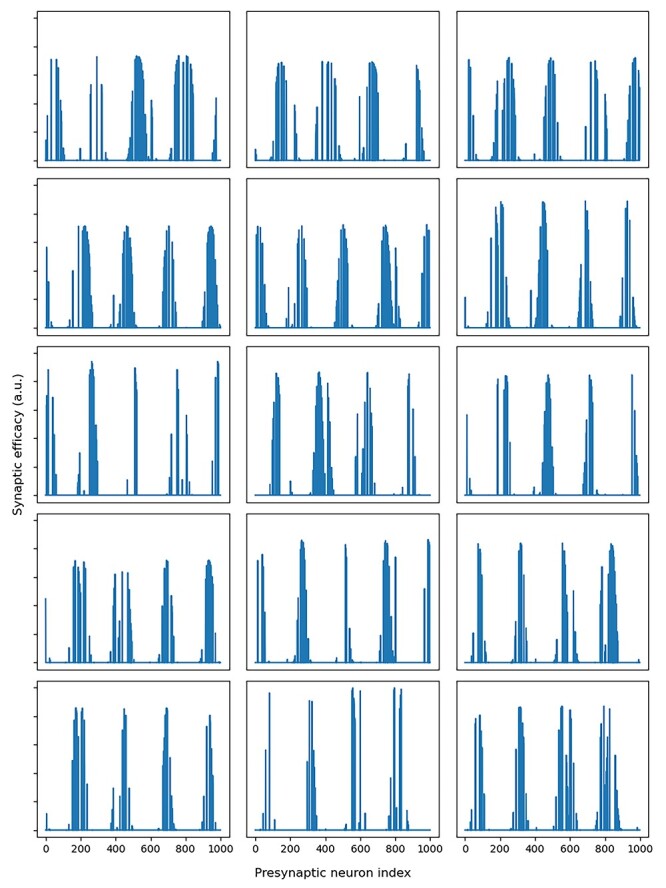
Synaptic weights VIS → STATE for the top 15 STATE cells shown in Figure 4, recorded immediately after the training phase. The model is trained with the agent rotating within the environment with five idealized distal visual landmarks present. In the neural network model, the egocentric bearing to each of the five distal landmarks is represented by a ring of 200 visual cells, denoted VIS in Figure 1*b*, where each visual cell is tuned to a different egocentric bearing within [0, 360] degrees. This gives a total of 1000 VIS cells, which are represented along the abscissa of each of the subplots. We observe that, as a result of competitive learning during training, each STATE cell has developed strong connections from local clusters of visual cells representing particular egocentric bearings to each of the distal landmarks. With competitive learning, when a STATE cell becomes activated due to the agent being oriented in a particular HD during training in the light, the cell learns to respond to all of the VIS inputs that are currently coactive. This means that each cell learns to respond to a set of preferred egocentric bearings to the distal landmarks that are consistent with each other in the sense that they correspond to the same single HD. This, in turn, ensures that each STATE cell effectively responds to the HD corresponding to its set of preferred egocentric bearings to the distal landmarks. The pattern of learnt connection strengths shown here is responsible for the kind of HD tuning observed in Figure 4. Each plot shows multiple peaks, as there are multiple (5) distal landmarks visible; since they are at infinite distance, there is a perfect correlation in the agent’s bearing to each, and the competitive learning therefore does not learn a preference for particular landmarks.

To confirm that this directional selectivity amongst the STATE cells is not an artifact of the testing regime, we interrogate the learned synaptic connectivity from the visual input cells (representing the egocentric bearings to the five distal visual landmarks) to the STATE cells (that have developed HD responses). In Figure 5, we observe that, after training, each STATE cell has developed strong connections from subpopulations of visual cells representing particular egocentric bearings to each of the five distal visual landmarks. Since the distal landmarks remain static within the environment, this pattern of synaptic efficacies ensures that the STATE cells are effectively tuned to respond to particular HDs. That is, the learned response properties of the STATE cells are a direct consequence of these learned patterns of synaptic efficacies from the visual input cells. Note that we do not impose any topological ordering on the STATE cells: this Gaussian-like tuning is entirely self-organized and a consequence of the Gaussian tuning of the VIS cells. Note also that the cells do not learn a preference for any landmark (or VIS subpopulation) over any other, since the five landmarks are never occluded and being placed at infinite distance, their bearings are perfectly correlated.

### Distal Landmarks are Required for Learning HD Representation

#### Study 2

Next, we investigate the effects of varying the kinds of visual landmarks present within the environment on the self-organization of the neural network architecture shown in Figure 1*b*, which receives self-motion inputs from AHV cells. We simulate the operation of this model within environments that contain either distal visual landmarks, or proximal visual landmarks, or both distal and proximal visual landmarks, as shown in the left panel of Figure 3. Note that, unlike in study 1, we now use ecological distal landmarks, visible beyond the boundary of the navigable environment but not at infinite distance, in order to conform to experimental conditions, under which landmarks cannot be placed at infinite distance. We explore how the kinds of visual landmarks present affect the nature of the spatial representations that emerge amongst the STATE cells during training. For these simulations, the model was trained with the agent both translating and rotating within the environment according to a random walk. We included 18 landmarks in each simulated environment: either 18 proximal or 18 distal, or 9 proximal plus 9 distal.

In the Introduction, we hypothesized that the self-organization of the neural network model using associative learning rules would be guided by correlated sensory information in the visual and idiothetic signals. We proposed that such correlated sensory signals would drive the network to learn to represent a particular form of spatial state such as HD or place.

**Figure 6:**
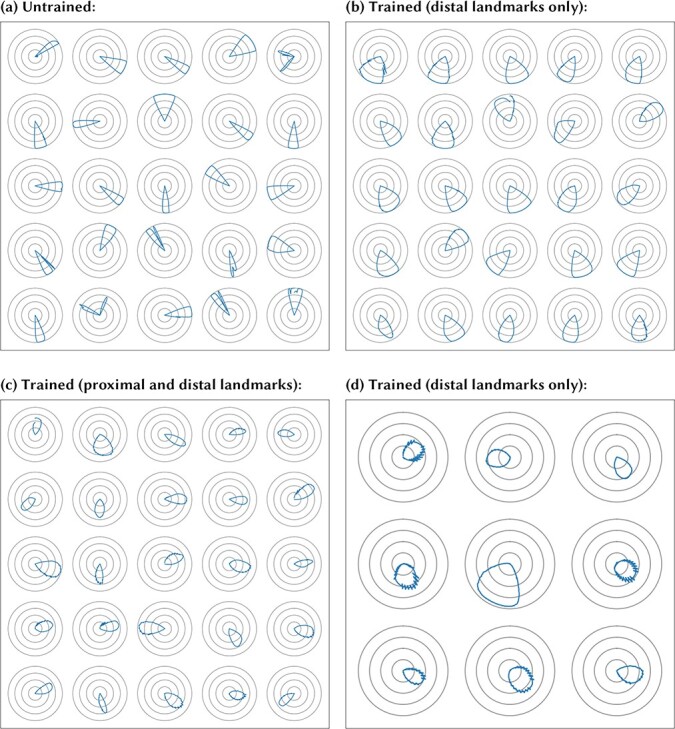
Simulation of STATE cell responses in the HD cell model shown in Figure 1*b* with AHV self-motion inputs. For those simulations in which the model was trained, the model was trained with the agent both translating and rotating within the environment according to a random walk. Each of the four plots in the figure shows the HD tuning of several randomly selected STATE cells under different training conditions, averaged over locations in the environment (see Methodology for testing details). Top left (*a*): network is untrained. We observe some HD preference induced by the random initial synaptic weights, but note that this tuning is irregular and sometimes split into multiple peaks. Top right (*b*): after training the network with only ecological distal visual landmarks (beyond the boundary of the navigable maze but not at infinity). Each of the STATE cells has learned to respond selectively to a single, localized interval of the HD space, like real HD cells found in the rat brain. Bottom left (*c*): after training with both distal and proximal visual landmarks present. The STATE cells have again learned to respond to single, localized regions of the HD space, like real HD cells in the brain. The STATE cells have succeeded in developing HD cell responses anchored to the distal landmarks regardless of the presence of proximal landmarks. Bottom right (*d*): out of the whole population of 1000 STATE cells, we only find nine cells with weak HD selectivity. These do not cover the whole circle, and display a degree of instability in their responses not otherwise observed when distal visual landmarks are present. This demonstrates the importance of distal landmarks for the emergence of HD responses.

In the case of HD, the heading of the agent is encoded in the visual signal representing the egocentric bearings to distal visual landmarks, and the changes in these bearings to distal landmarks are correlated with the AHV self-motion signal. Associative learning in the synaptic connections is able to exploit this correlation to drive the development of STATE cells that are tuned to respond to particular HDs. If instead the agent only has a visual input representation of the egocentric bearings to proximal visual landmarks, then changes in these egocentric bearings are not consistently correlated with AHV signal. For example, if the agent passes in a straight line (without rotation) beside a proximal visual landmark, this will cause the visual representation of its egocentric bearing to sweep through a half-turn in the absence of any AHV signal representing a change in HD. Therefore, we hypothesized that in an environment with only proximal visual landmarks, the model will not develop a HD representation.

Figure 6 shows the results of four different simulations as follows: the network is untrained (*a*, top left), the network is trained with only distal visual landmarks (*b*, top right), the network is trained with only proximal visual landmarks (*d*, bottom right), and the network is trained with both proximal and distal visual landmarks (*c*, bottom left). For each of these simulations, Figure 6 shows the tuning of several randomly selected STATE cells in the HD space. As described in the methodology, the tuning was computed by recording the STATE cell responses while the agent rotated in each direction (clockwise and anticlockwise according to each available AHV) at each location of a grid discretizing the environment, and then averaging the cell responses over locations andHDs.

In the untrained case, Figure 6*a* (top left) shows that the initial random synaptic weights induce a slight HD selective tuning in some STATE cells, but that this is uneven and irregular: quite unlike the clear encoding shown in Figure [fig:hd], which we therefore conclude was indeed learned.

The case of training with only distal visual landmarks (Fig. 6*b*, top right) is similar to that shown in Figure 4 except with the agent now performing a random walk in the 2D environment during training. It can be seen that each of the STATE cells has learned to respond selectively to a single, localized interval of the HD space, like real HD cells found in the rat brain.

However, when the model is trained with only proximal visual landmarks (Fig. 6*d*, bottom right), the model does not develop a sufficient HD representation in which many STATE cells mimic the responses of HD cells in the brain; indeed, we find that fewer than 1% of the STATE cells have developed any HD selectivity.

**Figure 7:**
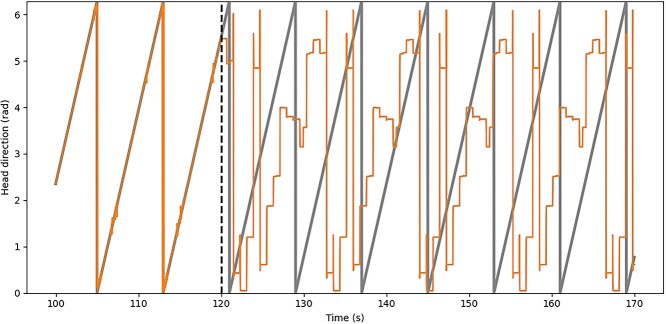
Simulation of path integration in the HD cell model shown in Figure 1*b* with AHV self-motion inputs. The model is first trained with the agent moving (translating and rotating) within the environment in the light with a mixture of proximal and distal visual landmarks present. After training, the model is tested with the simulated agent rotating on the spot initially in the light but later in the dark. In this situation, the simulated agent receives self-motion (idiothetic) input signals representing the AHV of the agent, but the visual input is eliminated halfway through the simulation. The figure shows the HD decoded from the layer of STATE cells (orange line), as well as the true HD (solid gray line). The vertical black dashed line indicates the moment of inactivation of the visual input signal. It is evident that after inactivating the visual input, the model is able to continue to update its internal representation of HD using the self-motion AHV signals with some degree of accuracy. Although there is some drift in the estimated HD, we hypothesize that this follows from the small cell population sizes used in the model.

Where the visual signal contains information about the egocentric bearings to both proximal and distal visual landmarks (Fig. 6*c*, bottom left), competitive learning enables the network to develop an HD representation, with only slight weakening in comparison with the distal only condition. Thus, the model displays robust behavior in the presence of a mixture of proximal and distal visual landmarks.

### The HD Model Performs Path Integration

#### Study 3

Next, we demonstrate that the HD cell model shown in Figure 1*b* with AHV self-motion inputs has learned to perform path integration; that is, to update its internal representation of HD from idiothetic AHV self-motion signals in the absence of visual input.

The model is first trained with the agent translating and rotating within the environment in the light with a mixture of proximal and distal visual landmarks present. After training the network, we record the population activity of the STATE cells and STATE × ACT cells during rotation on the spot. The visual input is kept active for the first part of the simulation, but switched off halfway through. To decode the HD signal in a biologically plausible manner, we learn a linear regression model after the training phase (see methodology for details).

In Figure 7, we plot the HD signals decoded from the STATE cells representing HD (orange curve) before and after inactivation of the visual input. We also plot the true HD (gray curve). Despite the jumpiness of the transitions, we find that, on average, the model is able to perform reasonably accurate path integration, with an angular head rotation speed of 71% of the true value. Here, we used 1000 STATE cells and 2000 STATE × ACT cells. We conjecture that a substantially larger network and/or longer training time may improve the accuracy of the path integration (see Discussion).

### FS × HD Idiothetic Input Generates a Place Representation

#### Study 4

In this study, we simulated the place cell model shown in Figure 1*c*, where the self-motion cells (represented by the ACT cells in Fig. 1*a*) contain two subpopulations of cells representing either FS or HD. We show that the general model shown in Figure 1*a* can develop place cells when we switch the self-motion signal from AHV to a combination of FS and HD. Mathematically, this combination encodes planar velocity. Whereas AHV is the time derivative of HD, planar velocity is the time derivative of planar location.

In these simulations, the HD self-motion input to the place cell model was computed artificially as a shifting Gaussian profile on an idealized ring of HD cells in order to represent the current HD of the agent, rather than using the HD representation learned by the HD model shown in Figure 1*b*. In a future study, these two neural network models will be linked so that they develop both HD cells and place cells simultaneously.

The model is first trained with the agent undertaking random exploration (i.e., translating and rotating) through the environment in the light. During training, all of the synaptic connection weights within the model are self-organized using the learning rules described in the Methods section. After training, the model is tested by traversing the environment systematically along a Cartesian grid lattice and recording the STATE cell responses at each location (see methodology for details).

**Figure 8:**
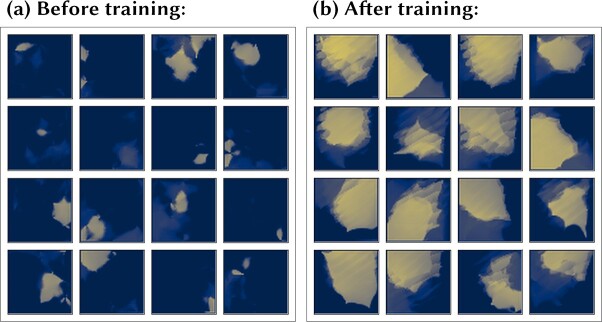
Simulation of the place cell model shown in Figure 1*c* with the self-motion cells (designated ACT in Fig. 1*a*) comprised of two subpopulations of cells representing either FS or HD. The model is first trained with the agent moving randomly (i.e., translating and rotating) within the environment in the light with only proximal visual landmarks present. During training, all of the synaptic connection weights within the model are self-organized using the learning rules described in the Methods section. After training, the model is tested by rotating the agent through a full circle in clockwise and anticlockwise directions at each location in the (discretized) environment, and then averaging the response over HDs at each location. The left panel shows the place response of 16 randomly selected STATE cells before training; the right panel shows the response of the same cells after training. We observe the characteristic location selectivity of place cells, with individual STATE cells displaying a single place field covering a unique portion of the environment. That is, each STATE cell responds selectively to a localized region of the environmental space, with different STATE cells responsive to different places. These STATE cells thus display the typical response properties of place cells. Although, as in Study 2, we see in the left panel evidence of the early preferences induced by the random initial synaptic weights, we note that place selectivity is not consistently evident in the untrained responses, demonstrating that the responses observed on the right were indeed learned.

Figure 8 shows simulation results from the place cell model shown in Figure 1*c* in an environment with only proximal visual landmarks present. Each plot in the right panel shows the average response of one randomly selected STATE cell as the agent moves within the 2D environment during testing; in the left panel, the plots show the corresponding responses of the same cells before training. After training, these cells display responses typical of place cells found in the rat brain. That is, each STATE cell responds selectively to a localized region of the environmental space, with different STATE cells responsive to different places. Moreover, we note that these responses are learned: those shown in the left panel are comparatively weak and nonselective, though they do show some evidence of the preferences which will later be reinforced by competitive learning to form the basis of the responses on the right.

**Figure 9:**
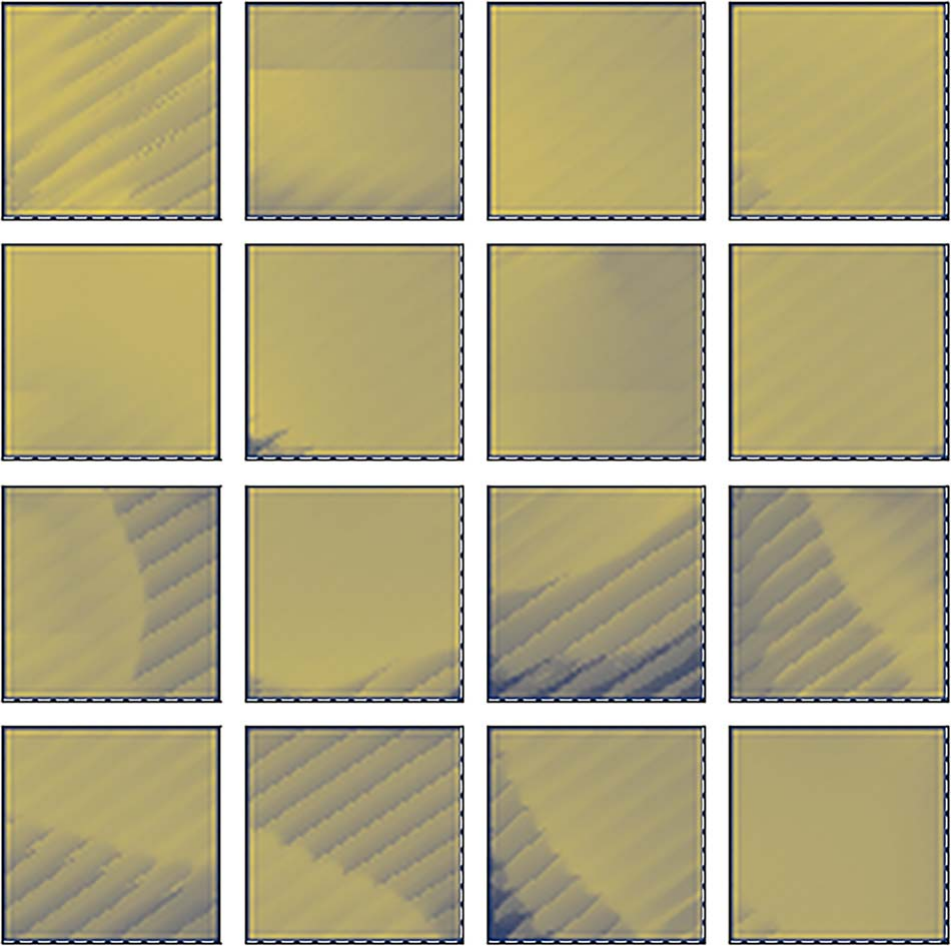
STATE cells in the place model, with only distal visual landmarks, do not develop place-specific responses. We present results of simulating the place cell model shown in Figure 1*c* with the self-motion action cells (designated ACT in Fig. 1*a*) comprised of two subpopulations of cells representing either FS or HD. The model is trained with the agent moving randomly (translating and rotating) within the environment in the light with only distal visual landmarks present. There are no proximal visual landmarks. After training, the model is tested by rotating the agent through a full circle in clockwise and anticlockwise directions at each location in the (discretized) environment, and then averaging the responses of individual STATE cells over HDs at each location. Results are shown for 16 STATE cells after training. With only distal visual landmarks, we do not observe the characteristic location selectivity of place cells. That is, the STATE cells do not respond selectively to a single, localized region of the environmental space. These STATE cells thus fail to display the typical response properties of place cells.

In comparison, Figure 9 shows simulation results from the place cell model shown in Figure 1*c* after training in an environment with only *distal* visual landmarks present. Each plot again shows the average response of a randomly chosen STATE cell as the agent moves within the 2D environment during testing. With only distal visual landmarks present, the STATE cells do not learn to display the characteristic response properties of place cells.

To summarize, as in the case of the HD cell model, we make two observations. Firstly, the place cell model learns a representation of the state—in this case, place—in accordance with the kinds of state transitions encoded by the self-motion (idiothetic) signals—in this case, FS × HD. Secondly, this learning requires that the visual input contain information about the state that is correlated with said state transitions. Contrary to the HD case, place transitions are correlated with changes in the egocentric bearings of the agent to the proximal visual landmarks: this is just the mathematics underlying triangulation. Therefore, in an environment with only distal visual landmarks, for which forward motion has no effect on the egocentric bearings to distal visual landmarks, the network cannot infer the place in which the agent is located from the visual input.

### HD Cells Do Not Display Response Characteristics of Place Cells

#### Study 5

Finally, we verify that the two different types of STATE cells, namely HD cells and place cells, which develop within their corresponding neural network models, do indeed have such distinct tuning. That is, we demonstrate that the STATE cells in the HD cell model shown in Figure 1*b* have HD tuning but not place tuning, while the STATE cells in the place cell model shown in Figure 1*c* have place tuning but not HD tuning. After training both the HD cell model and place cell model in the same environment with both proximal and distal visual landmarks, we investigate the learned response properties of the STATE cells. We find that STATE cells with HD tuning in the HD cell model do not have place-specificity, and conversely that STATE cells with place tuning in the place cell model only have weak HD-specificity.

The models in this study are trained with the agent moving randomly (i.e., translating and rotating) within the environment in the light with a mixture of proximal and distal visual landmarks present. After training, the model is tested as in the preceding study by traversing the environment systematically along a Cartesian grid lattice and recording the STATE cell responses at each location (see Methodology for details). To measure the HD tuning, we average over grid locations, whereas to measure the place tuning, we average overHDs.

**Figure 10:**
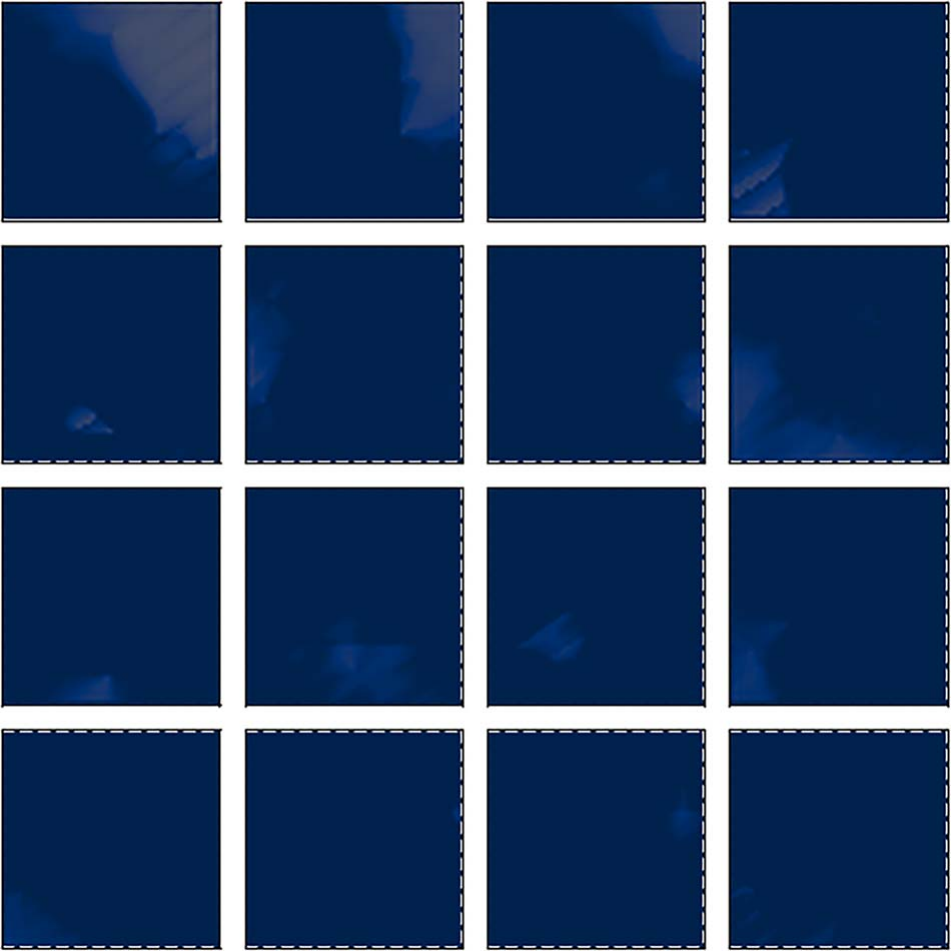
STATE cells in the HD model, with AHV idiothetic input, do not show place-specific responses. We present responses of the STATE cells in the HD cell model shown in Figure 1*b* with AHV self-motion inputs. The model is trained with the agent moving (i.e., translating and rotating) within the environment in the light with a mixture of proximal and distal visual landmarks present. After training, the model is tested by rotating the agent through a full circle in clockwise and anticlockwise directions at each location in the (discretized) environment, and then averaging the response over HDs at each location. Results are presented after training. We do not observe the characteristic location selectivity of place cells. That is, each STATE cell fails to respond selectively to a single localized region of the environmental space.

Figure 10 demonstrates that STATE cells in the HD cell model shown in Figure 1*b*, with AHV idiothetic input, do not show place-specific responses. Each plot shows the average response of one randomly chosen STATE cell. These cells clearly do not display the responses characteristic of place cells in the brain: their responses are largely homogeneous over the environment.

**Figure 11:**
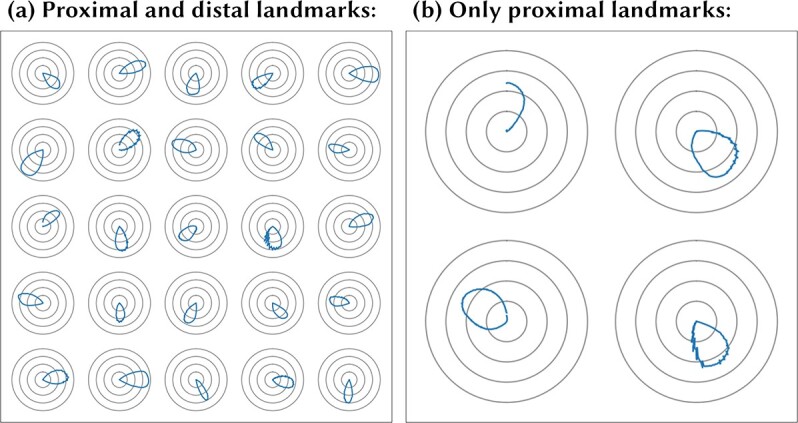
STATE cells in the place model, with FS × HD idiothetic input, encode weak HD information, and only with distal landmarks visible. We present results of simulating the place cell model shown in Figure 1*c* with the self-motion action cells (designated ACT in Fig. 1*a*) comprised of two subpopulations of cells representing either FS or HD. The model is trained with the agent moving (i.e., translating and rotating) within the environment in the light with a mixture of proximal and distal visual landmarks present. After training, the model is tested by rotating the agent through a full circle in clockwise and anticlockwise directions at each location in the (discretized) environment, and then averaging the responses of individual STATE cells over HDs at each location. The figure shows the tuning curves of several randomly selected STATE cells during testing (left: with both proximal and distal landmarks; right: with only proximal landmarks). We see that some STATE cells in the “place” model do exhibit some directional selectivity, but that this is comparatively weak. In the proximal-only case, we show the only four STATE cells (out of a population of 1000) found to have HD selectivity, again demonstrating the importance of distal landmarks. Some directional selectivity of place cells is in line with intuition (as the sensory input certainly contains directional information, which is transiently correlated with the agent’s rotation, even in the proximal-only case), and with experimental evidence (Muller et al. 1994). Note that, as in Figure 4, the discontinuity in the upper-left subplot is a sampling artifact.

Figure 11 shows that STATE cells in the place cell model shown in Figure 1*c*, with FS × HD idiothetic input, do not show strong HD responses. The figure shows the tuning curves of a number of selected STATE cells during testing. We find that, when the environment contains distal landmarks and the conjunctive cell input contains correspondingly correlated signals (in this case, an HD representation), then the STATE cells’ tuning does exhibit some weak directional selectivity, in accordance with experimental evidence (Muller et al. 1994). However, when the environment contains only proximal landmarks—which are therefore not highly correlated with the conjunctive cell input—we find that only 4 out of the entire population of 1000 STATE cells are HD-selective, despite most being place-selective. A small amount of directional selectivity is in line with our expectations, as the bearing representation that constitutes the sensory input certainly contains directional information that is transiently correlated with the agent’s action input (which has an HD component here); an interesting avenue for future experiments would be to compare the timescale of this correlation with the stability of the directional selectivity of place cells both simulated and biological.

## Discussion

In this paper, we have shown that the general CANN architecture shown in Figure 1*a* is able to develop either HD cells or place cells during visually guided learning as the simulated agent explores a 2D visual training environment containing proximal and distal landmarks. Whether HD cells or place cells develop depends critically on the kind of self-motion (idiothetic) input signals available to the model. It is an important feature of the model that all of the synaptic connections in the network self-organize during training using biologically plausible associative learning rules. Our model therefore provides theoretical justification and functional roles for empirically observed structures such as conjunctive cells and axonal conduction delays and their interaction with the developmental sensory environment, and thus makes predictions about the effect of disrupting these structures. We now review the implications of our results in this context, highlighting these empirical predictions and steps for futurework.

If the model shown in Figure 1*a* incorporates self-motion cells (designated ACT) that represent AHV then the STATE cells develop into HD cells. This special case of the network architecture is shown in Figure 1*b*. However, this requires that the training environment contains distal landmarks. The reason for this is that it is the change in egocentric bearing to the distal, not proximal, landmarks that is consistent with the AHV input signal. Because of this, the inputs representing the egocentric bearings to the distal landmarks and the AHV inputs are able to reinforce each other during self-organization of the synaptic connectivity within the neural network model during training leading to the development of STATE cells with response properties mimicking those of HD cells. Moreover, we showed that such HD cells develop strong afferent synaptic connections from visual neurons encoding a single, localized region of the space of egocentric bearings to a distal landmark. The HD cells thus inherit their firing properties by becoming anchored to the distal landmarks. The model behavior is robust in that HD cells still develop if the training environment contains a mixture of distal and proximal landmarks. In this case, the effect of the sensory input from the proximal landmarks washes out without affecting the nature of the spatial encoding (i.e., HD tuned cells) that develops. This is because the change in egocentric bearing to the proximal landmarks is not correlated with the AHV self-motion input signal.

If the model shown in Figure 1*a* incorporates self-motion cells (designated ACT) that represent a combination of FS and HD then the STATE cells develop into place cells. This special case of the network architecture is shown in Figure 1*c*. For this to occur, the training environment needs to contain proximal landmarks. This is because the change in egocentric bearing to the proximal, not distal, landmarks is correlated with the FS × HD input signals. Given this, there is mutual reinforcement between the visual inputs representing the egocentric bearings to the proximal landmarks and the FS × HD self-motion inputs during training leading to the development of STATE cells with the tuning of place cells. The model behavior is again robust in that place cells continue to develop if the training environment contains a mixture of distal and proximal landmarks. In this situation, the effect of the input from the distal landmarks washes out without affecting the kind of spatial encoding (i.e., place tuned cells) that develops. This is because the change in egocentric bearing to the distal landmarks is not correlated with the combined FS × HD self-motion input signal.

Our simulation results show that a single biologically plausible CANN architecture shown in Figure 1*a*, which self-organizes its synaptic connectivity using competitive learning during exploration of a training environment, is able to develop a representation of either HD or place depending on the nature of the self-motion signals available.

We also demonstrate that the HD cell model shown in Figure 1*b* is able to learn to update its internal representation of HD as the agent rotates in the dark—a property of the model known as “path integration.” The model is able to perform path integration at a reasonable level of accuracy, with an angular head rotation speed of 73% of the true value.

Path integration in the models relies on the presence of axonal transmission delays in the bidirectional connectivity between the STATE cells and STATE × ACT cells, as originally proposed by Walters et al. (2013). This suggests an adaptive role for such axonal transmission delays in the brain: delayed signals encode the recent past, and can be exploited by associative learning to learn the temporal state transitions required for path integration. Without this theoretical insight, axonal delays might otherwise seem like an irrelevant, and possibly even maladaptive, consequence of the physical constraints of neurobiology.

We hypothesize that accuracy of path integration in our model is related to the fidelity of the learned state transitions encoded in the synaptic connections to and from the layer of combination cells (denoted by the STATE × ACT cells in Fig. 1*a*), and that larger networks will improve this fidelity. Moreover, we conjecture that incorporating a range of axonal transmission delays on the bidirectional connectivity between the STATE cells and STATE × ACT cells is not only more biologically realistic, but also important for path integration performance: a range of delays would enforce consistency of the learned state transitions across a temporal window, rather than merely between two time points. Walters et al. (2013) have previously demonstrated a somewhat similar two-layer model of HD cells that was able to learn to perform path integration across synaptic connections with a distribution of different axonal transmission delays. The factors governing the accuracy of path integration have also been investigated by Page et al. (2015). They found that path integration accuracy is reduced by large rise times in the responses of neurons in the model, that is, the time it takes for neurons to start firing when they begin to receive excitatory input.

We have striven to develop neural network architectures, with neural dynamics and synaptic learning rules, which are broadly biologically plausible. However, in order to analyze the basic operation of these kinds of spatial processing models, we have implemented the minimal network architectures that will replicate the key behaviors of interest. It is therefore important to consider the correspondence between the spatial representations and structures of our idealized models and those known to exist in the brain.

Mapping our HD cell model onto the known physiology of the HD system in the rat brain is relatively straightforward. We propose that our STATE cells that develop into HD cells correspond to HD cells found in the LMN, and that our STATE × ACT cells that develop into HD × AHV cells correspond to HD × AHV cells found in the dorsal tegmental nucleus (DTN). DTN is known to receive AHV input (Bassett and Taube 2001), and to exhibit conjunctive HD × AHV responses (Chen et al. 1994; Sharp 1996; Stackman and Taube 1998; Sharp, Tinkelman, et al. 2001b; Bassett and Taube 2005; Jercog et al. 2019), such that its disruption impairs the entire HD system (Bassett et al. 2007). The two regions are known to be bidirectionally connected, although the connections from DTN to LMN are thought to be largely inhibitory (Wiener and Taube 2005). Consequently, a future development of our model will be to reproduce these results with inhibitory connectivity from the STATE × ACT cells to the STATE cells, and introduce some form of anti-Hebbian plasticity in these connections. As a result of this proposed mapping of our model onto the HD system in the rat brain, we predict that lesions to the DTN will disrupt the ability of LMN cells to disambiguate proximal from distal cue information, thereby destabilizing the HD representation, and will disrupt the animal’s ability to perform path integration; we expect similar disruption from selective impairment of DTN-LTN synapses. Because the conjunctive representation is distributed, we expect the disruption to be a monotonic function of the size of the lesion or impairment.

By contrast, mapping our place cell model directly onto hippocampal place cell circuits in the rat brain is less straightforward. Nevertheless, there are still strong parallels between the hippocampus and our place cell model. The hippocampus contains place cells that are tuned to respond when the rat enters localized areas within the environment (O'Keefe and Dostrovsky 1971; O'Keefe 1976). Moreover, some place cells are modulated by a combination of both FS and HD (Leutgeb et al. 2000; Sargolini 2006; Lu and Bilkey 2009; Chen et al. 2012). These cells appear to correspond to the combination cells, that is, PLACE × FS × HD cells, in our place cell model. However, the place cells and place cells modulated by FS and HD are mixed up in the same hippocampal field. This means that there will be bidirectional connections between the place cells due to the high level of recurrent connectivity (Rolls 2007). Page et al. (2015) have shown that introducing recurrent connections between the STATE cells in a CANN leads to inaccurate path integration, because the effect of these recurrent connections is to add an erroneous drag effect that slows down path integration. This suggests that the site of path integration of the rat’s location within the environment could be in another brain area such as entorhinal cortex (EC), where grid cells are found (McNaughton et al. 2006).

It is possible to take a broader view of the models presented in this paper, and in particular, the general CANN architecture shown in Figure 1. Our models sit in the context of recent evidence (Behrens et al. 2018) suggesting that navigation in apparently nonspatial environments, such as planning how to cook a meal, supervenes on the same mechanisms as navigation in explicitly spatial environments, such as mazes. The claim is then made that spatial navigation is evolutionarily prior, and so it is no surprise that the brain should make use of pre-existing mechanisms in novel environments (Garvert et al. 2017). Our results qualify this interpretation in the following way. A key part of the reason that “abstract navigation” supervenes on spatial navigation mechanisms is that both are instances of inference in some latent state space. We started with a simple one-dimensional case—HD—and subsequently generalized to two-dimensional place. But there is no reason a priori to stop there: it is easy to imagine a high-dimensional state of which physical location and heading form mere subspaces, especially when ecologically valid activities contain complex subtasks. For instance, whilst cooking, an agent must navigate the physical space of the kitchen, as well as the abstract space of the process of combining the ingredients of the meal. Even in such a highly abstract case of navigation, the underlying structure is the same: transitions in a latent state space induced by action. Our results show that solving this problem makes only minimal demands of neural circuitry.

In future work, we will make a number of improvements to the biological realism of the models. Firstly, the self-motion (idiothetic) input signals used in our models are highly simplified: whereas here we have used a binary code for the self-motion inputs, the corresponding self-motion cells in the brain have a smoother tuning—see Bassett and Taube (2001) for the case of AHV cells. Future work will extend the models by incorporating more realistic self-motion input cells, and in doing so, enable us to model path integration with the agent performing more realistic motions through the environment with smoothly varying velocities. A further extension will be to combine the HD cell model and place cell model into a single unified model, in which the STATE cells representing HD in the former model provide the HD component of the self-motion inputs to the latter model. We will investigate whether such a unified model can simultaneously self-organize both HD cells and place cells, and learn to perform path integration in both of these subsystems. Finally, we hypothesize that a similar model to the present place cell model will demonstrate the emergence of grid cells (Hafting et al. 2005), which are also hypothesized to have an attractor network architecture (Rennó-Costa and Tort 2017) under conditions that force the learned representations to be highly compressed. We expect that, as has been previously reported (e.g., by Rennó-Costa and Tort 2017), the incorporation of grid cells into the place model will facilitate path integration.

In conclusion, this paper has presented a self-organizing general state space model shown in Figure 1, which is built on biological principles. We have shown how these principles lead to the emergence of HD cells and place cells in the context of different idiothetic signals. We showed that successful learning depends on the existence of correlations between the external sensory and internal idiothetic signals. This means that the environment must be sufficiently rich: HD cell development requires distal landmarks, whereas place cell development requires proximal landmarks. The competitive learning mechanism is sufficiently powerful to disambiguate the two kinds of spatial representations: the model was able to learn either of the representations given visual signals representing a mixture of distal and proximal landmarks. Finally, our model shows that biological features that may seem maladaptive—such as axonal transmission delays—may be exploited for functional reasons such as path integration. In this case, the delays are crucial for learning the state transitions for path integration. These features of our models, including the emergence of HD and place representations, combine together in the brain to support navigation within complex spatial environments containing proximal and distal landmarks.

## Funding

Oxford Foundation for Theoretical Neuroscience and Artificial Intelligence (OFTNAI).

## Notes

The authors would like to thank Daniel Walters and Hector Page for helpful discussions. *Conflict of Interest*: None declared.

## Supplementary Material

supplement-v1_tgab052
