# Supplementary material for: The Role of Idiothetic Signals, Landmarks, and Conjunctive Representations in the Development of Place and Head-Direction Cells: A Self-Organizing Neural Network Model
Source: Cereb Cortex Commun. 2021 Aug 27;3(1):tgab052. doi: 10.1093/texcom/tgab052 (PMC8763244; doi:10.1093/texcom/tgab052)

Supplementary Figures

Cross-environment response preservation

We took the proximal-landmark-only environment simulated in Study 4 (top-left below), and stretched it along the east-west axis, doubling its width accordingly, while retaining the relative positions of the landmarks within the environment (top-right below); mathematically, this deformation corresponds to a change of coordinate system. We then ran the same place-selectivity test as in Study 4, and plotted the place responses of the same 16 cells shown in the manuscript (reproduced bottom-left below). These responses are shown in the bottom-right panel of the figure below. We note that these place fields in most cases correspond qualitatively to transformations of the originally-developed fields (left), in agreement with the experimental evidence.

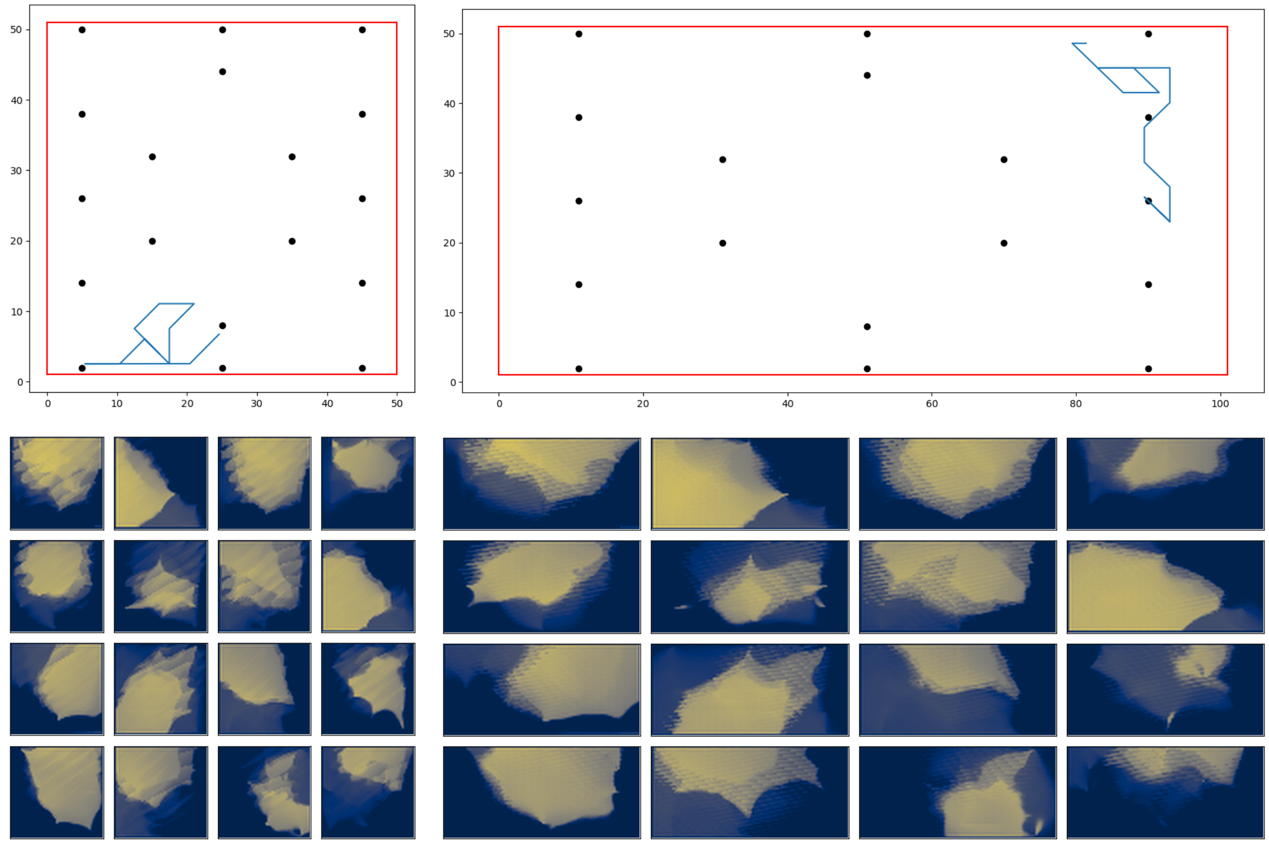

Border cells

In the figure below, we plot the place responses of a number of cells in the place-cell model from Study 4 (with proximal landmarks only) that resemble “border cells”.

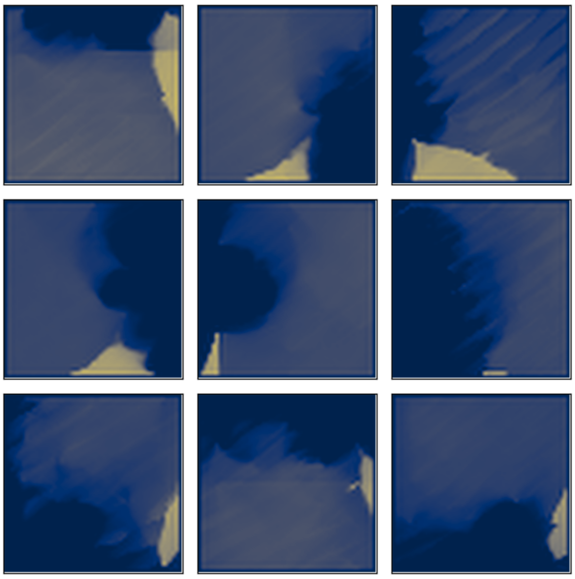

Supplement: supplement-v1_tgab052 [file supplement-v1_tgab052.pdf]
